# The fungal myosin I is essential for *Fusarium* toxisome formation

**DOI:** 10.1371/journal.ppat.1006827

**Published:** 2018-01-22

**Authors:** Guangfei Tang, Yun Chen, Jin-Rong Xu, H. Corby Kistler, Zhonghua Ma

**Affiliations:** 1 Institute of Biotechnology, Key Laboratory of Molecular Biology of Crop Pathogens and Insects, Zhejiang University, Hangzhou, China; 2 Department of Botany and Plant Pathology, Purdue University, West Lafayette, Indiana, United States of America; 3 Department of Plant Pathology, University of Minnesota, St. Paul, Minnesota, United States of America; 4 State Key Laboratory of Rice Biology, Zhejiang University, Hangzhou, China; University of Melbourne, AUSTRALIA

## Abstract

Myosin-I molecular motors are proposed to function as linkers between membranes and the actin cytoskeleton in several cellular processes, but their role in the biosynthesis of fungal secondary metabolites remain elusive. Here, we found that the myosin I of *Fusarium graminearum* (FgMyo1), the causal agent of Fusarium head blight, plays critical roles in mycotoxin biosynthesis. Inhibition of myosin I by the small molecule phenamacril leads to marked reduction in deoxynivalenol (DON) biosynthesis. FgMyo1 also governs translation of the DON biosynthetic enzyme Tri1 by interacting with the ribosome-associated protein FgAsc1. Disruption of the ATPase activity of FgMyo1 either by the mutation E420K, down-regulation of FgMyo1 expression or deletion of FgAsc1 results in reduced Tri1 translation. The DON biosynthetic enzymes Tri1 and Tri4 are mainly localized to subcellular structures known as toxisomes in response to mycotoxin induction and the FgMyo1-interacting protein, actin, participates in toxisome formation. The actin polymerization disruptor latrunculin A inhibits toxisome assembly. Consistent with this observation, deletion of the actin-associated proteins FgPrk1 and FgEnd3 also results in reduced toxisome formation. Unexpectedly, the FgMyo1-actin cytoskeleton is not involved in biosynthesis of another secondary metabolite tested. Taken together, this study uncovers a novel function of myosin I in regulating mycotoxin biosynthesis in filamentous fungi.

## Author summary

The mycotoxin deoxynivalenol (DON) is the most frequently detected secondary metabolite produced by *Fusarium graminearum* and other *Fusarium* spp. To date, relatively few studies have addressed how mycotoxin biosynthesis occurs in fungal cells. Here we found that myosin I governs translation of DON biosynthetic enzyme Tri1 *via* interacting with the ribosome-associated protein FgAsc1. Moreover, the key DON biosynthetic enzymes Tri1 and Tri4 are mainly localized to the toxisomes derived from endoplasmic reticulum under toxin inducing conditions. We further found that the FgMyo1-actin cytoskeleton was involved in toxisome formation but not for the biosynthesis of another secondary metabolite tested. Taken together, these results indicate for the first time that myosin I plays critical roles in mycotoxin biosynthesis.

## Introduction

Fusarium head blight (FHB) caused predominately by *Fusarium graminearum* is an economically devastating disease of small grain cereal crops [1]. This disease not only reduces yield and seed quality but also poses a great risk to human and animal health owing to its ability to contaminate grains with mycotoxins. The common mycotoxins associated with *F*. *graminearum* are deoxynivalenol (DON), nivalenol (NIV) and zearalenone (ZEA) [2]. Among them, DON is the most frequently detected mycotoxin in cereal grains throughout the world [3]. DON can inhibit protein synthesis by binding to the ribosome, and cause emetic effects, anorexia and immune dysregulation as well as growth, reproductive and teratogenic effects in mammals [4]. To minimize human and animal exposure to DON, regulatory organizations have established maximum permissible levels for DON in cereals and their products in many countries [5, 6]. However, DON contamination has become a challenging social issue because of the increased frequency and severity of FHB epidemics [7, 8].

DON contamination is closely linked to the severity of FHB disease in the field. The best way to prevent DON contamination would be to manage FHB in the field during crop cultivation. Currently, application of chemical fungicides is still a major approach against *F*. *graminearum* infection due to the lack of highly resistant wheat cultivars. However, application of several commercialized fungicides at sub-lethal concentrations could trigger DON biosynthesis [3, 9–11]. Recently, a novel cyanoacrylate fungicide phenamacril (JS399-19) has been marketed for FHB management and sale of phenamacril in China was approximately $40 million in 2016–2017. Interestingly, this small molecule compound (S1 Fig) exhibits highly specific antifungal activity against mycelial growth of a few *Fusarium* species including *F*. *graminearum*, *F*. *asiaticum*, *F*. *verticillioides* and *F*. *oxysporum* but not other fungal pathogens [12]. It shows excellent efficacy in controlling FHB in field trials [12, 13]. Combining inferences from genetic and biochemical results, we recently discovered that this compound acts on a novel target, the class I myosin (FgMyo1) in *F*. *graminearum*, which is homologous to Myo3p and Myo5p in *Saccharomyces cerevisiae* [12]. FgMyo1 is essential for *F*. *graminearum* growth. At the beginning of this study, we found that phenamacril not only suppressed the mycelial growth of *F*. *graminearum*, but also significantly inhibited DON production. These preliminary results suggested that the myosin I might also be involved in the secondary metabolism. Class I myosins are widely expressed, single headed and membrane-associated members of the myosin superfamily that participate in regulating membrane dynamics and structure in nearly all eukaryotic cells [14, 15]. However, the underlying function of myosin I in mycotoxin biosynthesis was totally unknown.

Enzymes for secondary metabolite synthesis may be compartmentalized at conserved sub-cellular sites in fungi, potentially channeling precursors, sequestering intermediates and products from the rest of the cell, thus promoting the efficiency of biosynthesis pathways [16]. In *Penicillium chrysogenum*, the major facilitator-type secondary transporter PenM promotes translocation of isopenicillin N from the cytosol to the peroxisomal lumen where it could be further metabolized to penicillin [17]. In *Aspergillus*, aflatoxin biosynthetic enzymes flow from peroxisomes to the motile vesicles termed aflatoxisomes in which aflatoxin biosynthesis takes place [18]. In *F*. *graminearum*, and other *Fusarium* spp, the biosynthetic pathway leading from the isoprenoid intermediate farnesyl pyrophosphate to DON involves 15 genes encoding the biosynthetic enzymes, a DON transporter and regulatory proteins, which are located on different chromosomes: the 25 kb *Tri5* cluster containing 12 genes on chromosome 2, the *Tri1-Tri16* locus with two genes on chromosome 1 and the single gene locus for *Tri101* on chromosome 3 [19–21]. Recent studies suggested that there is a cellular compartmentalization of biosynthetic enzymes for DON biosynthesis in *F*. *graminearum* [22]. Hydroxymethylglutaryl (HMG) CoA reductase (Hmr1) is a key enzyme in the mevalonate pathway for generating farnesyl pyrophosphate and indispensable for DON production. Fluorescent labeled Hmr1-GFP localized to the reticulate peripheral and perinuclear endoplasmic reticulum (ER) in toxin non-inducing conditions, while the ER was remodeled to form spherical and ovoid structures in the trichothecene biosynthesis inducing (TBI) conditions [16, 22]. In addition, the enzymes trichodiene oxygenase (Tri4) and calonectrin oxygenase (Tri1) catalyzing the early and late steps in the DON biosynthetic pathway were co-localized and showed the same localization patterns as Hmr1 in TBI medium [22, 23]. These novel cellular structures containing DON biosynthesis enzymes were named "*Fusarium* toxisomes" (“toxisomes” in shorter form in this study) [16, 22, 23]. However, the molecular mechanism of toxisome formation remains elusive.

The object of this study was to uncover the underlying mechanism of a myosin I inhibitor in regulating DON biosynthesis. Our results showed that myosin I plays critical roles in the translation of a Tri enzyme and in toxisome formation in *F*. *graminearum*. The importance of myosin I in the development of the mycotoxin biosynthetic machinery in *F*. *graminearum* may apply to other toxigenic pathogens.

## Results

### The trichothecene biosynthetic enzymes are localized at toxisomes under toxin inducing conditions

**Fig 1:**
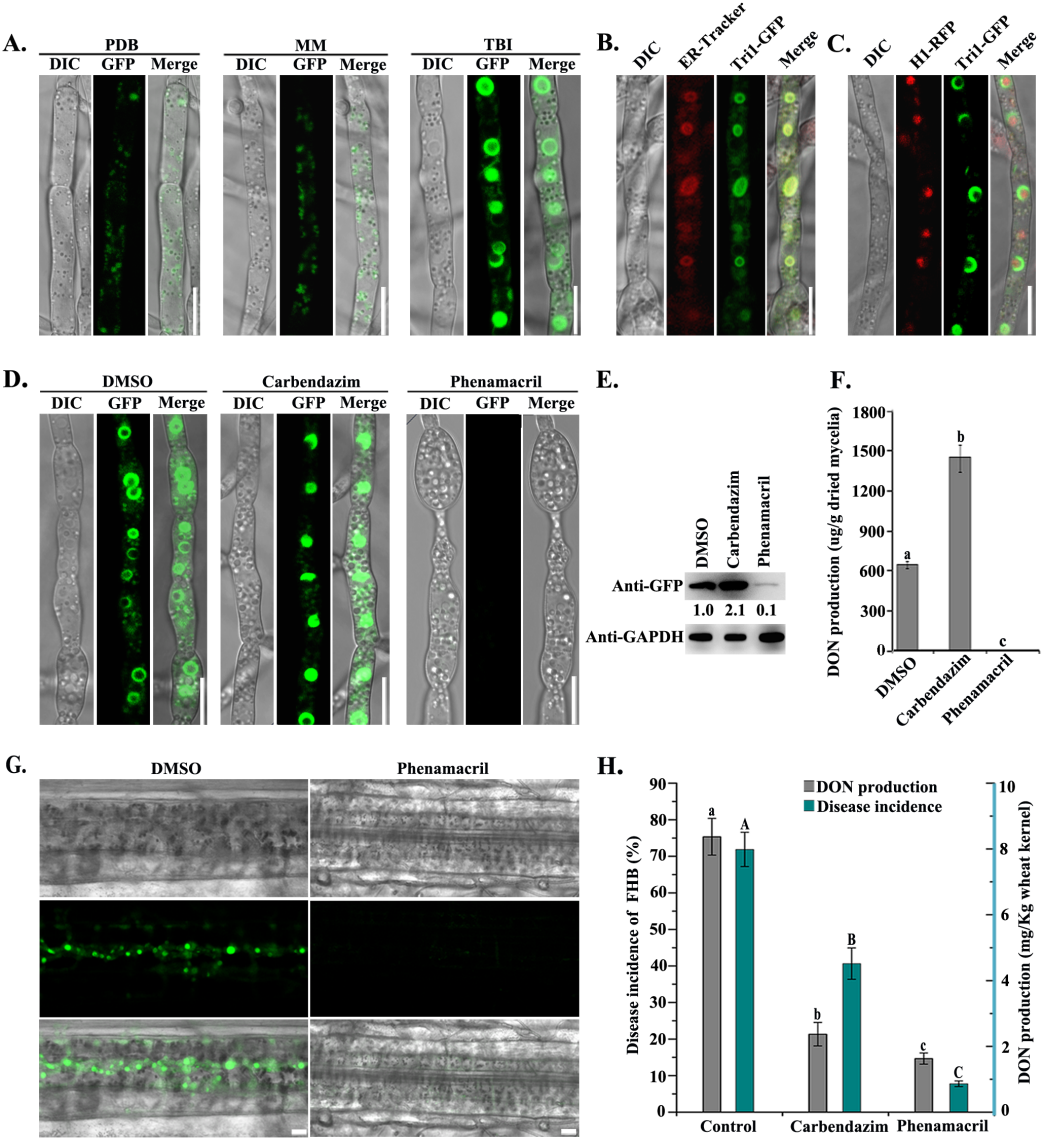
Phenamacril disrupted toxisome formation and subsequently inhibited DON production. **(A)** Tri1-GFP localized to the spherical structures (termed as toxisomes) in hyphae grown in TBI but not in PDB or MM. Images were taken after each strain was incubated at 28 °C for 48 h. Bar = 10 μm. DIC indicates differential interference contrast. **(B)** After growth in TBI for 48 h, hyphae of ΔTri1::Tri1-GFP were stained with the ER-tracker red and examined for GFP and ER tracker signals. Bar = 10 μm. **(C)** After growth in TBI for 48 h, hyphae of PH-1::Tri1-GFP+H1-RFP were examined for the co-localization of H1-RFP and Tri1-GFP. Bar = 10 μm. **(D)** Hyphae of ΔTri1::Tri1-GFP were treated with 0.5 μg/ml phenamacril, or 1.4 μg/ml carbendazim for 24 h in TBI before examination for GFP signals. The solvent DMSO was used as a control. Bar = 10 μm. (**E)** Western blots of proteins isolated from the same set of samples used in 1D were detected with the anti-GFP or anti-GAPDH antibody. **(F**) DON production was assayed for the wild-type PH-1 growth in TBI supplemented with 0.5 μg/ml phenamacril or 1.4 μg/ml carbendazim. The solvent DMSO was used as a control. Values on the bars followed by the same letter are not significantly different according to a Fisher’s least significant difference (LSD) test at *P* = 0.05. (**G)** Phenamacril inhibited toxisome formation in hyphae of ΔTri1::Tri1-GFP inoculated on wheat leaf. **(H)** Efficiencies of phenamacril (375 g/ha) and carbendazim (750 g/ha) in controlling Fusarium head blight (FHB) and DON contamination in the field trials. Values on the bars for disease incidence or DON production followed by the same letter are not significantly different according to a Fisher’s least significant difference (LSD) test at *P* = 0.05.

*TRI1* encodes calonectrin oxygenase that catalyzes calonectrin to 7, 8-dihydroxycalonetrin, which is a late step of DON biosynthesis in *F*. *graminearum* [24]. To characterize expression patterns and the sub-cellular localization of Tri1 protein under various conditions, the *TRI1* open reading frame tagged with GFP (green fluorescent protein) was introduced into a ΔTri1 *F*. *graminearum* PH-1 background, and the complemented strain expressing the Tri1-GFP (ΔTri1::Tri1-GFP) was used in the following study. In the toxin non-induction minimal (MM) or potato dextrose broth (PDB) media, Tri1-GFP displayed faint signals and was mainly associated with cell endomembrane (Fig 1A, left and middle panels). Tri1-GFP was highly induced and localized at the spherical structures (toxisomes) after 48 hours of incubation in the trichothecene biosynthesis induction (TBI) medium (Fig 1A right panel; S2 Fig) and *in planta* (Fig 1G, left panel). In addition, ER (endoplasmic reticulum)-tracker red staining indicated that Tri1-GFP was mainly localized to the ER in TBI cultures (Fig 1B), which is consistent with a previous finding that the toxisomes were identified as reorganization of the endoplasmic reticulum [22]. To determine whether the spherical structures were associated with the nucleus, we visualized nuclei by tagging the histone1 protein encoded by the FGSG_10800 locus with red fluorescent protein (RFP), which was designated as H1-RFP in the PH-1::Tri1-GFP strain. The H1-RFP/Tri1-GFP dual labeled strain was grown in TBI for 48 h, and localization of H1-RFP with Tri1-GFP was examined. As shown in Fig 1C, Tri1-GFP surrounded the H1-RFP labelled nuclei when the strain was cultured in the TBI medium. Moreover, the trichodiene oxygenase (Tri4) catalyzing the early step of DON biosynthesis had the same localization pattern as Tri1 (S3 Fig). Taken together, several lines of evidence suggested that trichothecene biosynthetic enzymes were clustered and localized to toxisomes derived from ER under the toxin inducing conditions.

### The myosin I inhibitor restrains toxisome formation and mycotoxin biosynthesis

Since the toxisomes are important for DON biosynthesis, a compound disrupting the toxisome formation may be very well effective against DON biosynthesis. To test this hypothesis, we established a "toxisome formation inhibitor screening" assay to quickly screen active compounds for their ability to restrict toxisome formation (see Material and methods). Briefly, the reporter strain expressing Tri1-GFP was grown in 24-wells plates supplemented with TBI medium. After 24 h incubation, individual compounds were added to wells. After incubation for another 24 h, the fluorescent intensity in each well was scanned with the plate-reader for the first round of screening. The wells with low or no fluorescent signals were further observed by microscopy. A total of 131 compounds including 11 commercial fungicides were tested for their activity against toxisome formation. Phenamacril was found to be the most efficient compound to inhibit toxisome formation and DON production (S4B and S4C Fig). The Tri1-GFP fluorescent signals were reduced dramatically and no typical toxisomes were observed in the mycelia treated with 0.5 μg/ml (approximately EC_90_ against mycelial growth) phenamacril for 6 h (S5B Fig) or 24 h (Fig 1D) in comparison with those in the non-treatment control. In addition, the beta-tubulin inhibitor, carbendazim, did not inhibit toxisome formation (Fig 1D). As shown in Fig 1E, the translation levels of Tri1-GFP were further verified by immunoblot assay using an anti-GFP antibody. Consistent with the microscopic observation, the intensity of the Tri1-GFP band from the strain treated with carbendazim increased more than 2-fold as compared with the non-treated control. In contrast, a faint immunoblot band was detected in the same strain treated with phenamacril (Fig 1E). Correspondingly, DON in the mycelia treated with phenamacril was below the level detectable by LC-MS (liquid chromatography-mass spectrometer) (Fig 1F). Furthermore, we tested the efficiency of phenamacril against DON production *in planta* and in the field. As shown in Fig 1G, phenamacril also clearly inhibited toxisome formation in hyphae of *F*. *graminearum* inoculated on wheat leaf. In the field trials, this antifungal compound was very effective against FHB and DON production in comparison with the control chemical carbendazim (Fig 1H). The class I myosin (named FgMyo1) of *F*. *graminearum* has been identified as the target of phenamacril [12]. Taken together, these results strongly indicated that the myosin I inhibitor phenamacril was able to inhibit DON biosynthesis in *F*. *graminearum*.

### The myosin I is essential for toxisome formation

**Fig 2:**
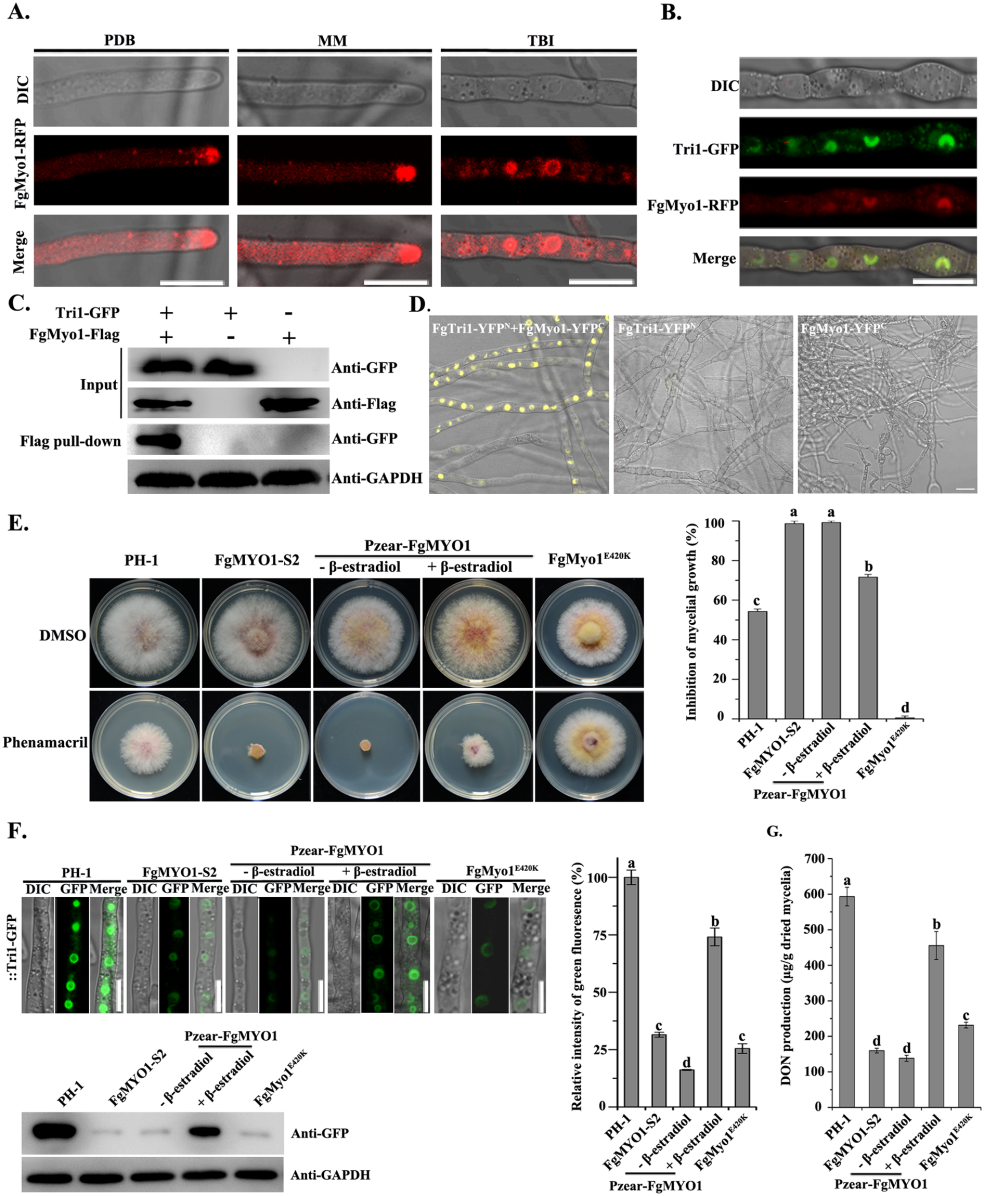
FgMyo1 is required for toxin biosynthesis. **(A)** Localization of FgMyo1-RFP in hyphae of PH-1::FgMyo1-RFP growth in toxin non-inducing media PDB and MM at 28°C for 48 h. Bar = 10 μm. **(B**) FgMyo1-RFP was co-localized with Tri1-GFP under the toxin inducing condition. Bar = 10 μm. **(C)** The interaction of FgMyo1 with Tri1 was confirmed by co-immunoprecipitation (Co-IP) analysis. Total proteins (input) extracted from the strain bearing FgMyo1-3×Flag and Tri1-GFP constructs or a single construct (FgMyo1-3×Flag or Tri1-GFP) were subjected to SDS-PAGE, and immunoblots were incubated with anti-Flag and anti-GFP antibodies, as indicated (Input panel). Each protein sample was pulled down using anti-Flag agarose and further detected with anti-GFP antibody (Flag pull-down panel). The protein samples were also incubated with the anti-GAPDH antibody as a reference. **(D)** The interaction of FgMyo1 with Tri1 was confirmed by bimolecular fluorescence complementation (BiFC) analysis. The constructs of pFgTri1-YFP^N^ and pFgMyo1-YFP^C^ were co-transformed into PH-1 to generate the strain FgTri1-YFP^N^+FgMyo1-YFP^C^. The strains bearing a single construct (FgMyo1-YFP^C^ or FgTri1-YFP^N^) were used as negative controls. The YFP signals in hyphae of each strain grown in the TBI medium were examined under a confocal microscope. Bar = 10 μm. **(E)** The sensitivity of FgMyo1 derived mutants towards phenamacril. The wild-type PH-1, FgMyo1 silencing mutant FgMyo1-S2, inducible mutant Pzear-FgMYO1, and the point mutation strain FgMyo1^E420K^ were incubated on PDA supplemented with 0.3 μg/ml phenamacril (left panel). For the inducible mutant, PDA was also added with (+) or without (-) the inducer 30 μg/ml β-estradiol. Mycelial growth inhibition of each strain by phenamacril was quantified (right panel). Values on the bars followed by the same letter are not significantly different according to a Fisher’s least significant difference (LSD) test at *P* = 0.05. **(F)** The toxisome formation patterns in FgMyo1 derived mutants. Each strain was grown in TBI, and images were taken after incubation for 48 h (left-upper panel). The accumulation of Tri1-GFP protein in each strain was determined by western blot assay with the anti-GFP antibody. The protein samples were also incubated with the anti-GAPDH antibody as a reference (left-lower panel). The intensities of GFP signals in each strain were also quantified. Values on the bars followed by the same letter are not significantly different according to a Fisher’s least significant difference (LSD) test at *P* = 0.05 (right panel). **(G)** The DON production of FgMyo1 derived mutants. DON was extracted from mycelia of each strain grown in TBI for 7 days. Values on the bars followed by the same letter are not significantly different according to a Fisher’s least significant difference (LSD) test at *P* = 0.05.

Given that the myosin I inhibitor significantly reduces DON biosynthesis, myosin I may be critical for toxisome formation. In order to verify this, we tagged FgMyo1 with RFP to determine its subcellular localization. In toxin non-induction media MM and PDB, FgMyo1-RFP protein was detected as diffuse fluorescent signal in the cytoplasm, mainly localized at hyphal tips (Fig 2A). However, in the TBI medium, most FgMyo1-RFP fluorescence accumulated in subapical spherical structures (Fig 2A, right panel). To determine whether myosin I was localized to the toxisomes, a strain labeled with FgMyo1-RFP and Tri1-GFP was constructed and cultured in TBI. As indicated in Fig 2B, both proteins were mainly co-localized at the toxisomes. Additionally, Co-IP and BiFC (Bimolecular Fluorescence Complementation) assays showed that FgMyo1 interacted with Tri1 in toxin inducing condition (Fig 2C and 2D). Affinity capture mass spectrometry (ACMS) was then used to identify interacting proteins upon toxin-induction conditions using the dual tagged protein ZZ-Tri1-Flag as the bait. In the ACMS assay, FgMyo1 was captured by Tri1 (S1 Table). Furthermore, ten of the 30 Tri1-interacting proteins were described previously [22] as components of the toxisome, including the three cytochrome P-450 enzymes Tri1, Tri4 and Tri11 as well as HMG-CoA reductase. These results indicated that FgMyo1 interacts with Tri1 and thus has the potential for involvement in toxisome formation.

To verify the role of FgMyo1 in toxisome formation, we used a knock-down approach because *FgMYO1* is an essential gene in *F*. *graminearum* [12]. First, we took the advantage of the RNA interfering (RNAi) pathway to induce *FgMYO1* silencing with hairpin RNA (hpRNA), which has been proven to be efficient in knockdown of mRNA expression for target genes in *F*. *graminearum* [25]. The recombinant plasmid pSilent-FgMYO1, designed for generating the hpRNA of an *FgMYO1* fragment (540 bp), was introduced into the wild-type PH-1. Predicting that transformants with reduced expression of *FgMYO1* may grow poorly on the medium supplemented with phenamacril, we screened for transformants with increased sensitivity towards this compound and then verified the *FgMYO1* expression level by reverse transcription-PCR. Among the 20 transformants tested, four showed increased sensitivity to phenamacril, and the expression levels of *FgMYO1* were decreased 65%-90% in these silencing transformants in comparison with the wild type. The FgMYO1-S2 transformant, having the lowest *FgMYO1* expression (10% of the parent strain PH-1), was selected for further characterization. It had normal growth rate on PDA but failed to grow on PDA supplemented with phenamacril at 0.3 μg/ml (approximately EC_50_ against mycelial growth) (Fig 2E). As expected, the toxisome formation indicated by Tri1-GFP was significantly impaired and only faint fluorescent signals were observed in the mycelia of FgMYO1-S2 harboring Tri1-GFP (Fig 2F). Next, we replaced the native promoter of *FgMYO1* with the zearalenone (ZEA)-inducible promoter (*P*_*zear*_) [26] to generate a transformant that conditionally expressed *FgMYO1*. The resulting transformant (termed as P_zear_-FgMYO1) without ZEA induction was unable to grow on PDA supplemented with 0.3 μg/ml phenamacril (Fig 2E). Consistently, this strain formed very faint toxisomes in TBI without the inducer as compared to the wild type (Fig 2F upper panel). The defects in mycelial growth and toxisome formation of P_zear_-FgMYO1 were partially recovered by adding the inducer β-estradiol (Fig 2E and 2F, upper panel). In addition, translation levels of Tri1-GFP protein in above strains quantified by the western blotting assay were consistent with fluorescent signals (Fig 2F, lower panel). All of the above mutants, whether constructed by silencing or conditional expression, revealed significantly reduced DON production in TBI (Fig 2G). Since DON is a critical virulence factor and plays a significant role in the spread of pathogen within host tissues [27–29], it follows that each of these strains was severely attenuated in virulence toward flowering wheat heads (S6 Fig). These results confirmed that FgMyo1 plays an important role in toxisome formation.

### The myosin I is indispensable for translation of Tri1

**Fig 3:**
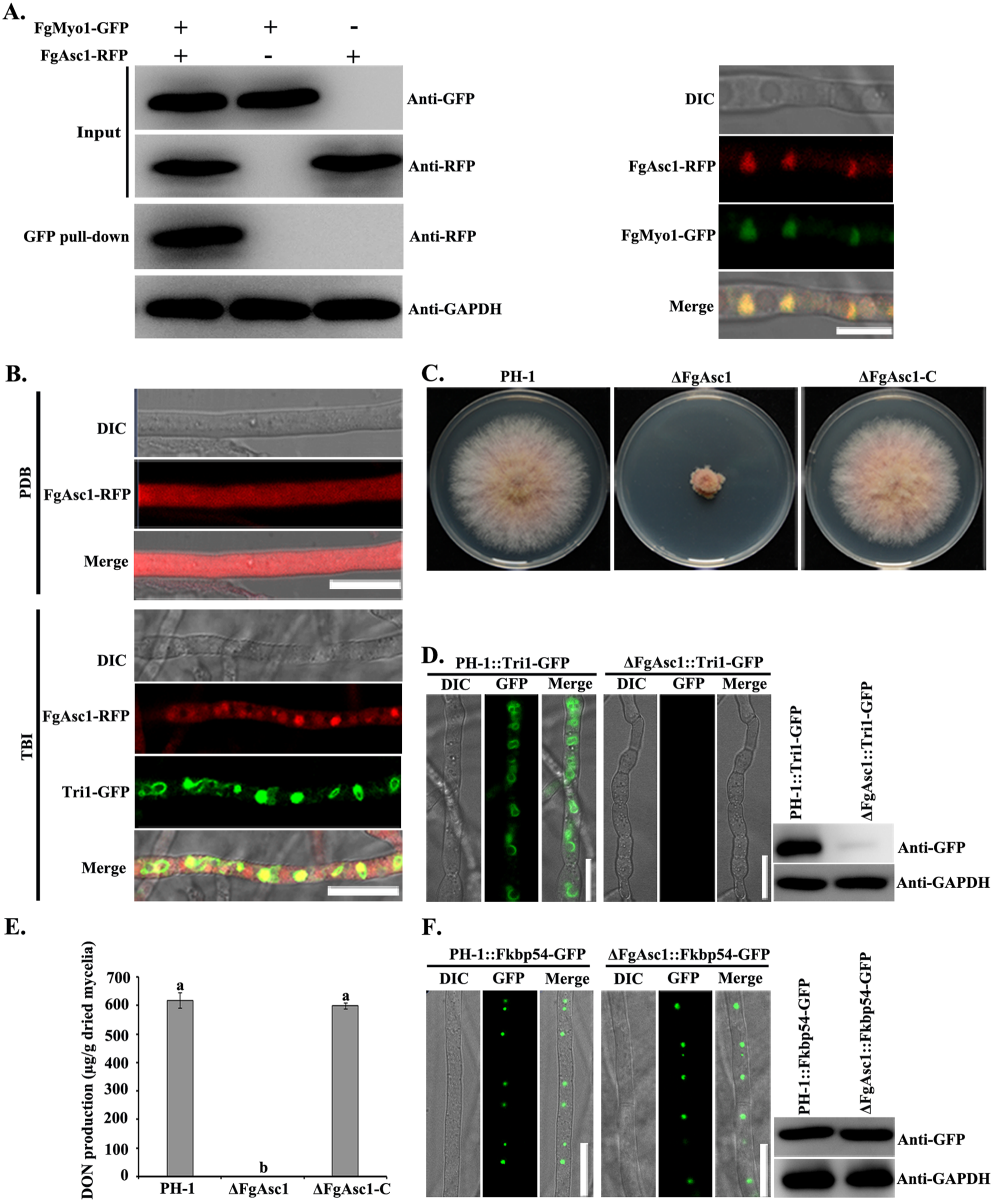
FgMyo1 regulates translation of Tri1 via interacting with the ribosomal protein FgAsc1. **(A)** The interaction of FgMyo1-GFP and Asc1-RFP was verified by the Co-IP assay (left panel). FgMyo1-GFP was co-localized with FgAsc1-RFP under the toxin inducing conditions. Bar = 10 μm. (**B**) Localization of FgAsc1-RFP in hyphae of PH-1::FgAsc1-RFP+Tri1-GFP grown in toxin non-inducing medium PDB (upper panel) or toxin inducing medium TBI (lower panel) for 48 h. Bar = 10 μm. (**C**) ΔFgAsc1 exhibited dramatically reduced hyphal growth on PDA. **(D)** Toxisome formation was not detected in ΔFgAsc1 grown in TBI medium (left panel). Bar = 10 μm. The accumulation of Tri1-GFP protein in ΔFgAsc1 was determined by a western blot assay with the anti-GFP antibody (right panel). **(E)** DON production was under a detectable level in ΔFgAsc1. Values on the bars followed by the same letter are not significantly different according to a Fisher’s least significant difference (LSD) test at *P* = 0.05. **(F)** Comparisons in localization (left panel) and translation level (right panel) of the FK506-binding protein Fkbp54 tagged with GFP in the wild type and in the ΔFgAsc1 mutant. Bar = 10 μm.

To gain an insight into the function of FgMyo1 in toxisome formation, we further conducted an ACMS assay using the dual tagged protein ZZ-FgMyo1-Flag as the bait. In the ACMS assay, the ribosome-associated protein Asc1 (hereafter named FgAsc1,) was captured by FgMyo1. Unexpectedly, FgAsc1 was also pulled down by Tri1 (S1 Table). In addition, the interaction of FgMyo1 and FgAsc1 was confirmed by Co-IP assay (Fig 3A, left panel), while the directly interaction between these two proteins was not verified by BiFC. Given that the translation level of Tri1-GFP protein was inhibited dramatically by phenamacril (Fig 1D), and Asc1 is a conserved ribosomal protein and is required for efficient protein translation [30,31], we inferred that FgMyo1 might regulate Tri1 translation *via* interacting with Asc1. To test this hypothesis, we first examined co-localization of FgMyo1-GFP and FgAsc1 tagged with RFP. In toxin non-inducing conditions, FgAsc1-RFP was detected as diffuse fluorescent signal in the cytoplasm (Fig 3B). However, in the TBI medium, most FgAsc1-RFP accumulated in spherical structures and co-localized with FgMyo1 (Fig 3A, right panel) and also with Tri1 at the perinuclear positions (Fig 3B, lower panel). Since Asc1 is ribosome-associated protein, to further visualize localization of ribosomes, FgRpL25 (an essential component of 60S subunit of ribosome [32, 33]) was tagged with mCherry under the control of its own promoter, and transformed into the wild type. Confocal microscopic examination showed that most FgRpL25-mCherry accumulated at the perinuclear positions in the toxin inducing conditions (S7 Fig, bottom panel). In contrast, FgRpL25- mCherry was mainly localized in the cytoplasm in the toxin non-inducing conditions (S7 Fig, upper panel). These results indicated that FgMyo1 interacts with the ribosome protein FgAsc1 in toxin inducing conditions.

To further understand the role of FgAsc1 in Tri1 translation, we constructed a deletion mutant of FgAsc1. As expected, the mutant exhibited dramatically reduced hyphal growth (Fig 3C, upper panel). The translation level of Tri1-GFP in this mutant was decreased markedly in comparison with that in the wild type (Fig 3D, right panel), and subsequently, toxisome formation and DON production was not detected in this mutant cultured in TBI medium (Fig 3D, left panel; Fig 3E). It is very interesting that the translation level of FK506-binding protein Fg_Fkbp54 (FGSG_01059) was not altered in ΔFgAsc1 as compared to that in the wild type (Fig 3F, right panel), suggesting that FgAsc1 controls the translation of some proteins (at least Tri1) but not all proteins, which is agreement with a role for Asc1 in regulating the translation of specific mRNAs in *S*. *cerevisiae* [31, 34]. Taken together, these results indicated that FgMyo1 was indispensable for translation of Tri1 protein by interacting with the ribosome protein FgAsc1.

### The myosin I-actin cytoskeleton participates in toxisome formation

**Fig 4:**
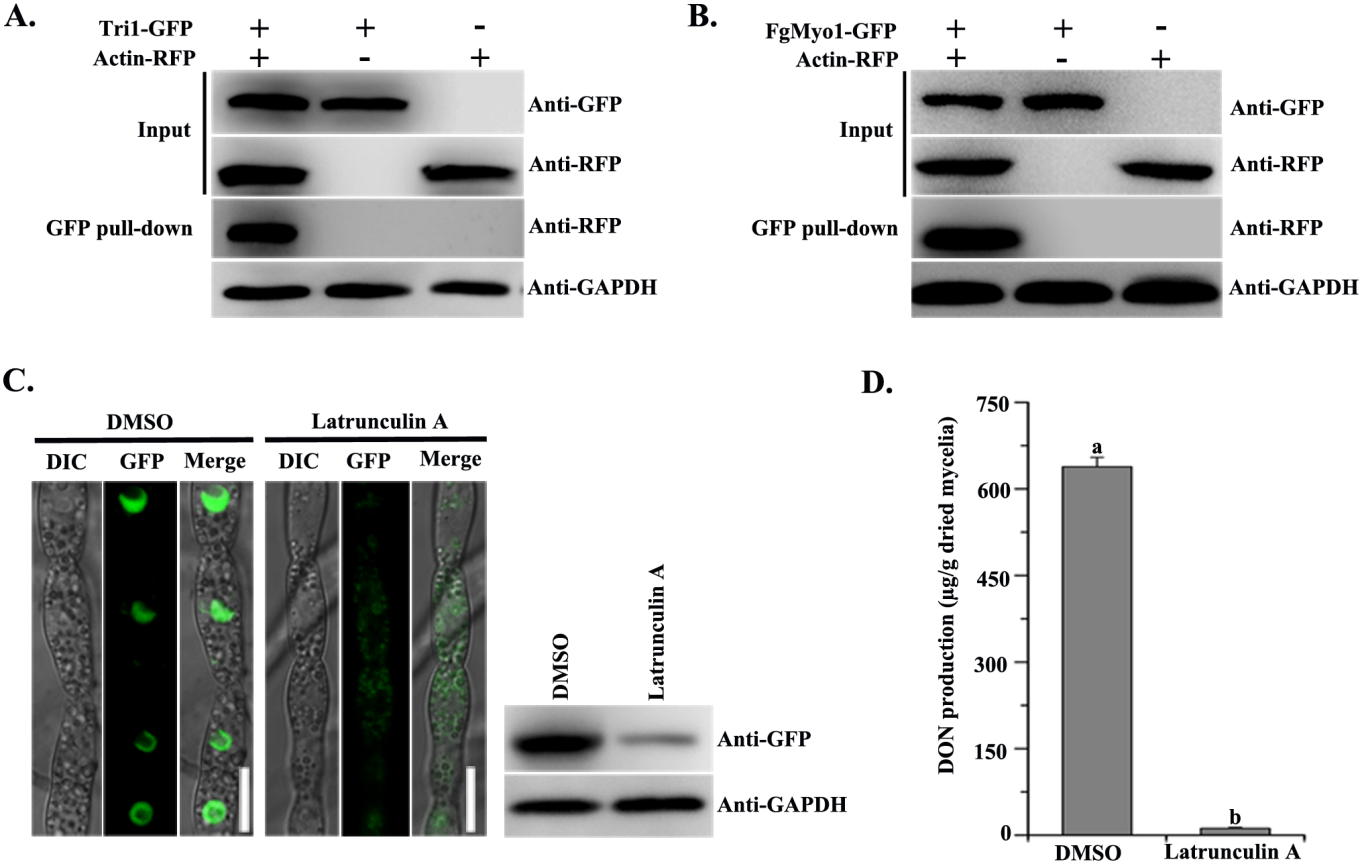
The actin cytoskeleton is involved in toxisome formation. **(A)** The interaction of Actin-RFP and Tri1-GFP was verified by the Co-IP assay. Total proteins (input) extracted from the strain bearing Actin-RFP and Tri1-GFP constructs or a single construct (Actin-RFP or Tri1-GFP) were subjected to SDS-PAGE, and immunoblots were incubated with anti-GFP and anti-RFP antibodies, as indicated (Input panel). Each protein sample was pulled down using anti-GFP agarose and further detected with anti-RFP antibody (GFP pull-down panel). The protein samples were also incubated with the anti-GAPDH antibody as a reference. **(B)** Co-IP analysis for verification of the interaction between FgMyo1-GFP and Actin-RFP. Total proteins (input) extracted from the strain bearing Actin-RFP and FgMyo1-GFP constructs or a single construct (Actin-RFP or FgMyo1-GFP) were subjected to SDS-PAGE, and immunoblots were incubated with anti-Flag and anti-GFP antibodies, as indicated (Input panel). Each protein sample was pulled down using anti-GFP agarose and further detected with anti-RFP antibody (GFP pull-down panel). The protein samples were also incubated with the anti-GAPDH antibody as a reference. **(C)** The actin polymerization inhibitor latrunculin A inhibited toxisome formation. After growth in TBI for 24 h, ΔTri1::Tri1-GFP was treated with 0.1 μg/ml latrunculin A for another 24 h before examination (left panel). The solvent DMSO was used as a control. Bar = 10 μm. The accumulation of Tri1-GFP protein was further verified by western blotting assay using the anti-GFP antibody (right panel). The protein samples were also incubated with the anti-GAPDH antibody as a reference. **(D)** DON was extracted from mycelia of PH-1 grown in TBI supplemented with 0.1 μg/ml latrunculin A. The solvent DMSO was used as a control. Values on the bars followed by different letters are significantly different according to a Fisher’s least significant difference (LSD) test at *P* = 0.05.

In a previous study, we found that the ATPase activity of FgMyo1 is dependent on actin. FgMyo1^E420K^ bearing a mutation at the actin interacting domain of FgMyo1, which caused the actin-activated ATPase activity of FgMyo1^E420K^ was reduced to 5% as that of the wild-type FgMyo1 [12]. Correspondingly, toxisome formation in this strain was markedly decreased in comparison with that of the wild type (Fig 2E, upper panel). Moreover, we found that components of the actin cytoskeleton were enriched in the ACMS with Tri1 and FgMyo1 as the bait (S1 Table), suggesting that actin cytoskeleton may be associated with toxisome formation in *F*. *graminearum*. To address this possibility, we further constructed a strain bearing actin-RFP and Tri1-GFP. Then, the interaction between actin-RFP and Tri1-GFP was further verified by Co-IP assay (Fig 4A). In *S*. *cerevisiae*, the myosin I interacts with the actin and is required for polarization of the actin cytoskeleton [35]. Consistent with what is known in *S*. *cerevisiae*, actin was also associated with FgMyo1 in the ACMS assay using FgMyo1 as the bait (S1 Table). In addition, the interaction of FgMyo1-GFP and actin-RFP was further confirmed by the Co-IP assay (Fig 4B).

Since actin is essential for *F*. *graminearum* growth, we were unable to obtain a knockout mutant of the *ACTIN* gene. Thus, to further investigate the function of actin in DON biosynthesis, the actin polymerization inhibitor latrunculin A was used to mimic impaired function of the actin cables. After treatment with latrunculin A at 0.1 μg/ml (approximately EC_90_ against mycelial growth of *F*. *graminearum*), the typical toxisome structures could not be observed, and Tri1-GFP was detected as diffuse fluorescent signal in the cytoplasm (Fig 4C, left panel). In addition, Tri1-GFP was noticeably decreased in the western blot assay upon latrunculin A treatment (Fig 4C, right panel). Subsequently, latruncunlin A showed strong inhibition of DON production (Fig 4D). These results indicated that the actin cytoskeleton is involved in toxisome formation in *F*. *graminearum*.

**Fig 5:**
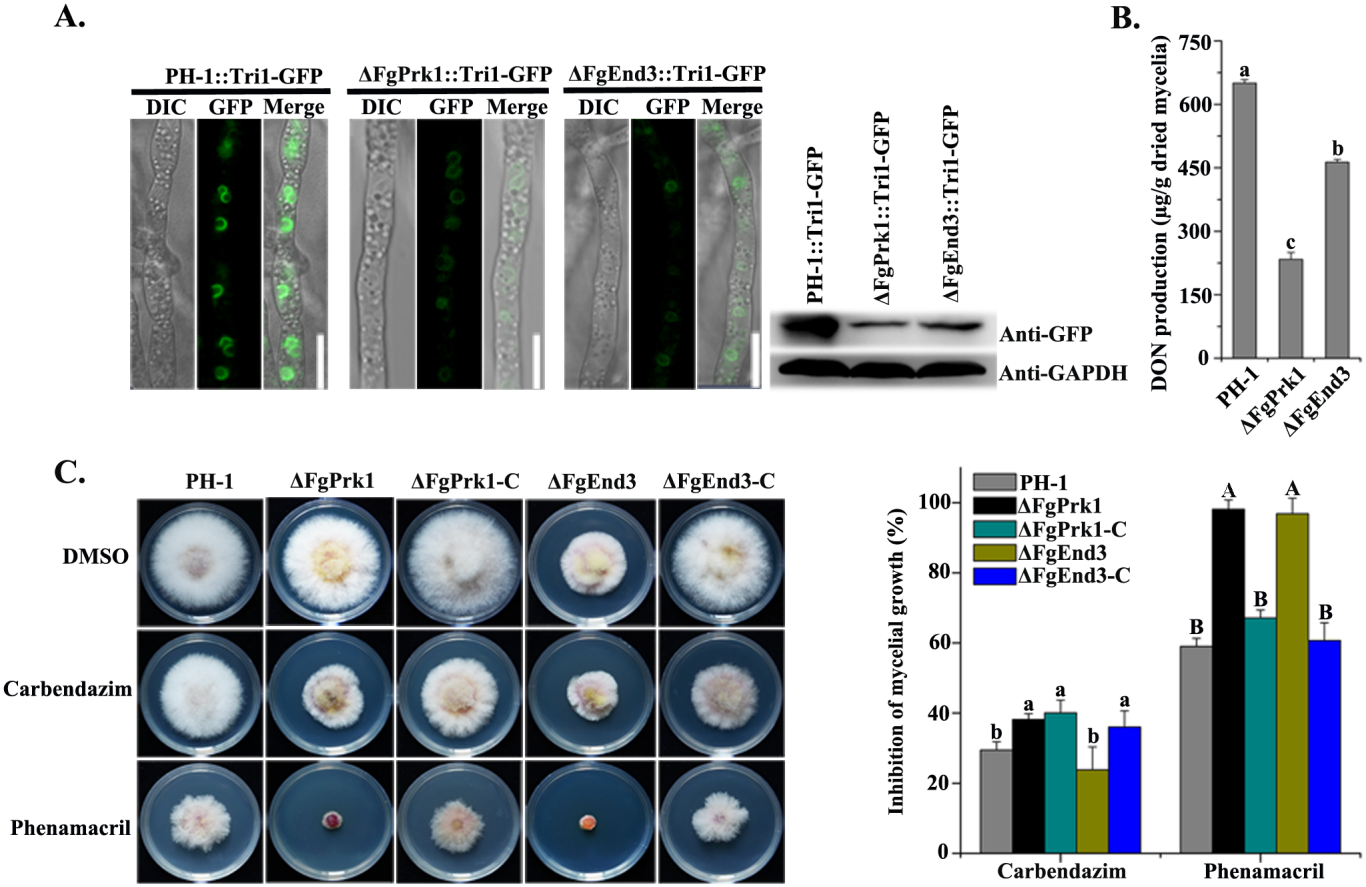
Deletion of the actin associated protein genes *FgPrk1* or *FgEnd3* hinders toxisome formation. **(A)** Toxisome formation in ΔFgPrk1 and ΔFgEnd3 (left panel). The images were taken after each strain bearing Tri1-GFP was incubated in TBI for 48 h. Bar = 10 μm. The accumulation of Tri1-GFP protein in each strain was determined by using a western blot assay with the anti-GFP antibody (right panel). The protein samples were also incubated with the anti-GAPDH antibody as a reference. **(B)** Production of DON in ΔFgPrk1 and ΔFgEnd3 after each strain was cultured in TBI for 7 days. **(C)** The sensitivity of ΔFgPrk1, ΔFgEnd3 and their complementation strains (ΔFgPrk1-C and ΔFgEnd3-C) towards phenamacril and carbendazim. Each strain was cultured on PDA supplemented with 0.3 μg/ml phenamacril or carbendazim (left panel). Mycelial growth inhibition of each strain by phenamacril or carbendazim was quantified (right panel). Values on the bars for each fungicide treatment followed by different letters are significantly different according to a Fisher’s least significant difference (LSD) test at *P* = 0.05.

In *S*. *cerevisiae*, Prk1 and End3 are involved in the organization of the actin cytoskeleton [36, 37]. To better understand the roles of the myosin I-actin cytoskeleton in toxisome formation, we therefore were interested in constructing deletion mutants of their orthologs FgPrk1 (FGSG_05586) and FgEnd3 (FGSG_09721). Toxisome formation in mycelia of these two gene deletion mutants harboring the tagged Tri1-GFP was examined. The Tri1-GFP signals decreased noticeably in both ΔFgPrk1 and ΔFgEnd3 mutants (Fig 5A, left panel). In addition, western blot assays confirmed the amount of Tri1-GFP protein in these mutants was considerably lower than that of the wild type under the toxin inducing condition (Fig 5A, right panel). Furthermore, these mutants produced significantly less DON as compared with the wild type (Fig 5B) and both mutants showed increased sensitivity to the myosin I inhibitor phenamacril (Fig 5C). Taken together, these results strongly indicated that the myosin I-actin cytoskeleton is essential for the toxisome formation in *F*. *graminearum*.

### The myosin I-actin cytoskeleton is not involved in pigmentation

**Fig 6:**
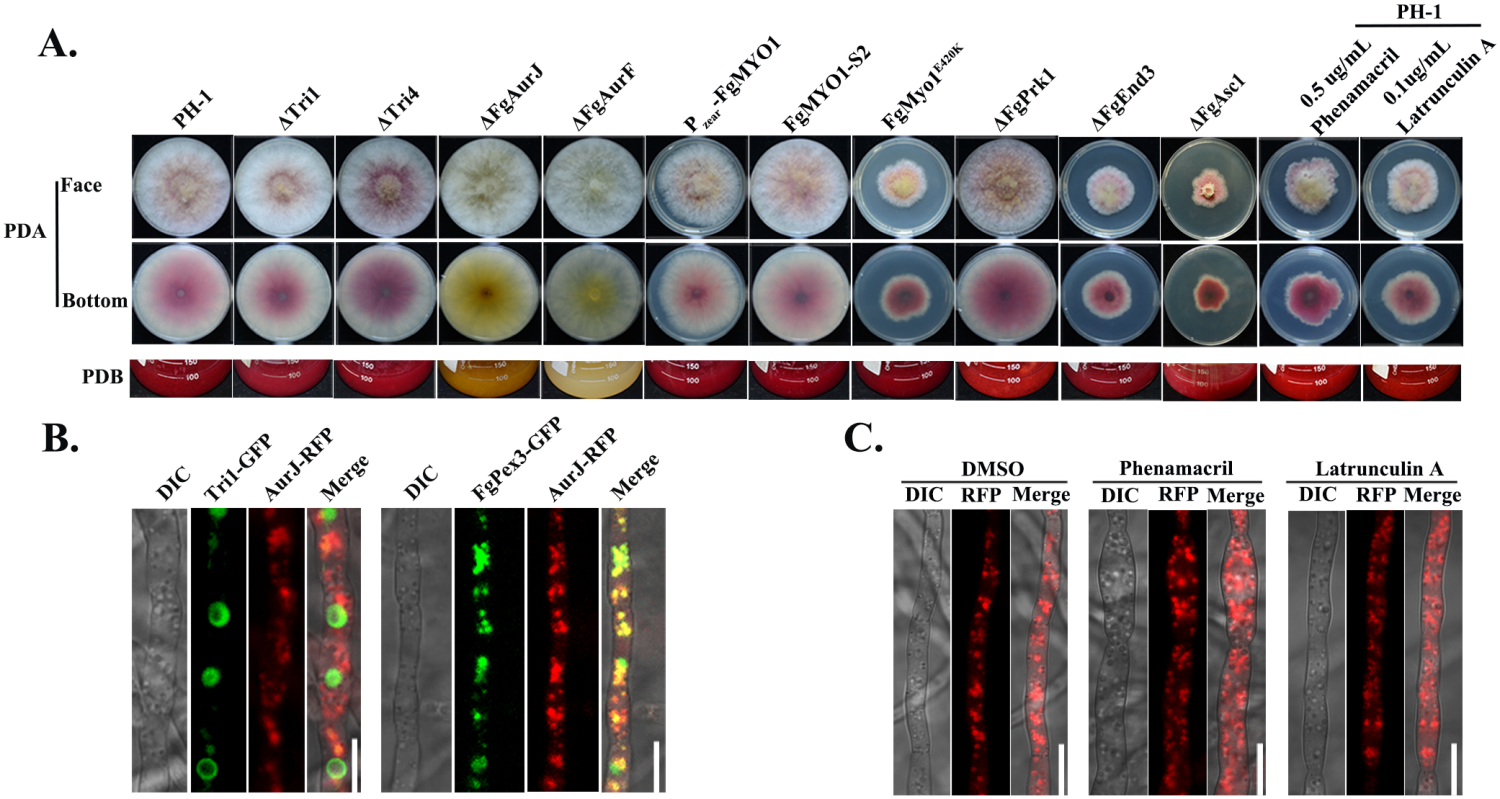
The myosin I-actin cytoskeleton is not associated with aurofusarin biosynthesis. **(A)** Comparisons of red pigment (aurofusarin) biosynthesis among the wild type and various mutants constructed in this study. Images were taken after each strain was grown on PDA or in liquid PDB. The myosin I inhibitor phenamacril and the actin polymerization inhibitor latrunculin A did not inhibit aurofusarin biosynthesis. (**B**) Co-localization analysis for Tri1-GFP or the peroxisome indicator FgPex3-GFP with the aurofusarin biosynthetic enzyme AurJ-RFP. A strain dual-labeled with either AurJ-RFP and Tri1-GFP or AurJ-RFP and FgPex3-GFP was grown in TBI for 48 h before observation. Bar = 10 μm. (**C**) Phenamacril and latrunculin A did not affect cellular localization of AurJ-RFP.

To test whether or not the myosin I-actin cytoskeleton is also necessary for biosynthesis of other secondary metabolites (SM), we examined aurofusarin biosynthesis because aurofusarin is a red polyketide pigment and easily visualized. As shown in Fig 6A, the FgMyo1 point mutation (FgMyo1^E420K^) and *FgMYO1* knockdown mutants had similar red pigmentation in comparisons with the wild type PH-1, as well as ΔTri1 and ΔTri4 mutants after incubation for 3 days on PDA or 5 days in liquid PDB. Consistent with these observations, phenamacril and latrunculin A did not inhibit aurofusarin biosynthesis in the wild type (Fig 6A). As controls, deletion mutants of aurofusarin biosynthesis genes AurJ and AurF did not produce the red pigment (Fig 6A). These results suggest that the myosinI-actin cytoskeleton is dispensable for aurofusarin pigmentation. To further confirm this finding, the aurofusarin biosynthesis gene AurJ was tagged with RFP and transformed into the wild type bearing Tri1-GFP or the peroxisomal structural protein FgPex3-GFP. As indicated in Fig 6B (left panel), AurJ-RFP was mainly located in the cytoplasm and presented in a punctuate pattern that was different from the Tri1-GFP localization. However, AurJ-RFP was clearly co-localized with FgPex3-GFP. These results indicate that aurofusarin might be synthesized in peroxisomes. In addition, the cellular localization and fluorescent intensity of AurJ-RFP was not discernibly affected by treatment with phenamacril or latrunculin A (Fig 6C). In summary, the myosinI-actin cytoskeleton is not involved in aurofusarin pigmentation in *F*. *graminearum*.

## Discussion

Trichothecenes are synthesized from acetyl-CoA as the basic precursor though the isoprenoid intermediate farnesyl pyrophosphate (FPP) and ultimately the trichothecene biosynthesis pathway [38]. The enzymes Tri1 and Tri4 are delivered to the specific cellular compartment known as the toxisome under the DON induction condition (Fig 1A, S2 Fig). This process is largely dependent on various environmental factors or stimuli, including nitrogen and carbon sources [10, 11, 39], amines [40], pH [41], light [42], and reactive oxygen species (ROS) [3]. Accumulating evidence indicates that some fungicides also stimulate DON biosynthesis. Milus and Parsons reported that propiconazole and tebuconazole treatments could result in a 50% increase in DON contamination in field trials [9]. Application of fluquinconazole or azoxystrobin reduced disease incidence on wheat spikes but led to a significant increase in DON production by *F*. *culmorum* or *F*. *graminearum* in the harvested grains [10]. The fungicides epoxyconazole and propiconazole could also stimulate DON production *in vitro* and in wheat grains [11]. Therefore, the effects for disease management by application of fungicides may not be consistent with the impacts on mycotoxin biosynthesis. In this study, we tested the effect of 131 antifungal compounds on DON biosynthesis and found that phenamacril showed significant inhibition against DON biosynthesis. In agreement with previous studies, other fungicides including the carbendazim and azoles at sub-lethal concentrations could stimulate DON biosynthesis. Therefore, the chemical fungicides for FHB management should be carefully considered to avoid stimulating mycotoxin biosynthesis.

In eukaryotic cells, myosins participate in a wide variety of cellular processes, including cytokinesis, organellar transport, cell polarization, transcriptional regulation, intracellular transport, and signal transduction [43, 44]. They bind to the filamentous actin or other binding partners, and produce physical forces by hydrolyzing ATP, therefore converting chemical energy into mechanical force [12–14, 44, 45]. The conserved head domain is accompanied by a broad diversity of N-terminal or C-terminal domains that bind to different molecular cargos, providing the functional specificity of myosin proteins [46]. A total of 31 defined myosin classes have been identified in eukaryotes based on genomic surveys and phylogenetic analyses [15,46]. Three myosins: an essential class II myosin FgMyo2 (FGSG_08719), a class V myosin FgMyo2B (FGSG_07469), and the essential class I myosin FgMyo1 (FGSG_01410) are recognized in *F*. *graminearum* [47]. FgMyo2 is specifically localized to the delimiting septum of phialides and conidia, and required for septation [48]. In addition, the expression levels of *TRI5* and *TRI6* were obviously higher in the FgMyo2B heterokaryotic disruption mutant than those in the wild type [16,49]. These studies indicated that FgMyo2 and FgMyo2B may not be involved in mycotoxin biosynthesis directly. In the current study, we found that FgMyo1 is necessary for toxisome formation. Moreover, we further proved that FgMyo1 was not essential for the biosynthesis of the polyketide secondary metabolite, aurofusarin. These data suggest that the myosin I, but not other myosin motors, participates in DON biosynthesis in *F*. *graminearum*.

The cellular compartmentalization (toxisome) for DON biosynthesis in *F*. *graminearum* was first described though the dynamic localization of fluorescent labeled Tri1 and Tri4 [23]. More recently, the toxisome was further identified as reorganization of the endoplasmic reticulum with pronounced expansion at perinuclear-and peripheral positions [22]. Consistent with that, results of the current study further confirmed that Tri1 and Tri4 are often localized in the perinuclear ER under the toxin inducing condition (Fig 1B), and that the ER was remodeled from thin reticulate ER (S8 Fig) in the toxin non-inducing conditions to thickened ER in the TBI conditions (Fig 1A). In addition, the ER remodeling is further supported by accumulation of the perinuclear ribosomes under the TBI conditions (S7 Fig) since ribosomes are often attached the rough ER.

The toxisome structures were predicted to confer multiple beneficial biological functions including clustering of DON biosynthetic enzymes, promoting the efficiency of DON biosynthesis, as well as serving as a self-protection system against the self-toxicity of the Tri products and reaction intermediates [9, 22]. To date, four proteins including Tri1, Tri4, Tri14 and Hmr1 were validated to be localized to toxisomes [16, 22, 23]. However, the molecular mechanism for the ER remodeling to toxisome remains unknown. In eukaryotic cells, structures and functions of ER are dynamically changed by various intercellular and extracellular stimuli. For example, the ER network of *Arabidopsis* undergoes extensive remodeling, which is critically depended on a myosin-actin cytoskeleton system [50]. The plant specific myosin XI provides the force to propel ER streaming and the dynamic rearrangement of the ER network depends on the propelling action of myosin-XI over actin coupled with a SYP73-mediated bridging [51]. Since *F*. *graminearum* doesn’t contain a myosin XI homologous protein, we infer that the FgMyo1-actin cytoskeleton may be involved in the ER remodeling for toxisome formation in *F*. *graminearum*. This inference is supported by multiple lines of evidence. First, FgMyo1 is comprised of the motor domain that binds to and interacts with actin [12, 18], an isoleucine and glutamine (IQ) motif, and a C-terminal tail. The tail domain contains a pleckstrin homology (PH) motif that is known to bind the anionic phospholipids in cellular membranes (S9 Fig) [52, 53]. The presence of a lipid-binding domain in the tail and an actin binding region in the motor domain equips the myosin I for cellular roles that link membranes to the actin cytoskeleton [54]. Second, dysfunction of FgMyo1 and actin by inhibitors disrupts the toxisome formation (Figs 1D and 4C). Third, knockdown expression of FgMyo1 or the deletion of actin cytoskeleton organization related genes FgPrk1 and FgEnd3 resulted in a defect in toxisome formation and a reduction in DON production (Figs 2F, 5A and 5B). Finally, the point mutation FgMyo1^E420K^ allowing only 5% of the wild-type ATPase activity also affected toxisome formation (Fig 2F), which is in agreement with the interpretation that the hydrolysis of ATP in FgMyo1 coverts the chemical energy into mechanical force and might provide the physical forces for ER remodeling.

In addition to providing the force for membrane dynamics, the myosin I motors have also been suggested to function as anchors or tethers between membranes and other proteins. In opossum kidney epithelial cells, Myo1b was found to tether amino acid transporters to the apical plasma membrane, thereby facilitating neutral amino acid transport across the membrane [55]. Similarly, Myo1a is important for the retention and localization of sucrose isomaltase in the intestinal brush border membrane [56]. Furthermore, the spatial association of nuclear myosin I with the ribosome protein S6 plays an important role in the export of small ribosomal subunits through the nuclear pores [57]. In current study, we found that FgMyo1 interacts with the ribosome-associated protein Asc1, thereby facilitating translation of toxin biosynthesis enzymes, and further contributing to toxisome formation in the toxin inducing conditions.

In eukaryotic cells, the myosin-actin system also plays important roles in endocytosis [58–60]. Consistent with that, deletion mutants of actin cytoskeleton organizing gene orthologs, Prk1 and End3 resulted in the defects in both endocytosis and toxisome formation in *F*. *graminearum*. However, the mutants of two conserved endocytic components (Apm4 and Abp1) still formed typical toxisomes in TBI (S10C Fig). Importantly, the FgMyo1^E420K^ mutant that exhibits the defect in toxisome formation (Fig 2F) retains the capability of endocytosis (S10A Fig), while the actin-activated ATPase activity of FgMyo1^E420K^ is very low (*circa* 5% as that of the wild-type FgMyo1) [12]. This finding is similar to a previous report that the Myo1 mutants of *Aspergillus nidulans* with no more than 1% of the actin-activated ATPase activity of wild-type Myo1 *in vitro* and no detectable *in vitro* motility activity can support fungal cell growth, albeit with a delay in germination time and a reduction in hyphal elongation [61]. Therefore, the myosin I mediated endocytosis process is not connected with the toxisome formation in *F*. *graminearum*.

**Fig 7:**
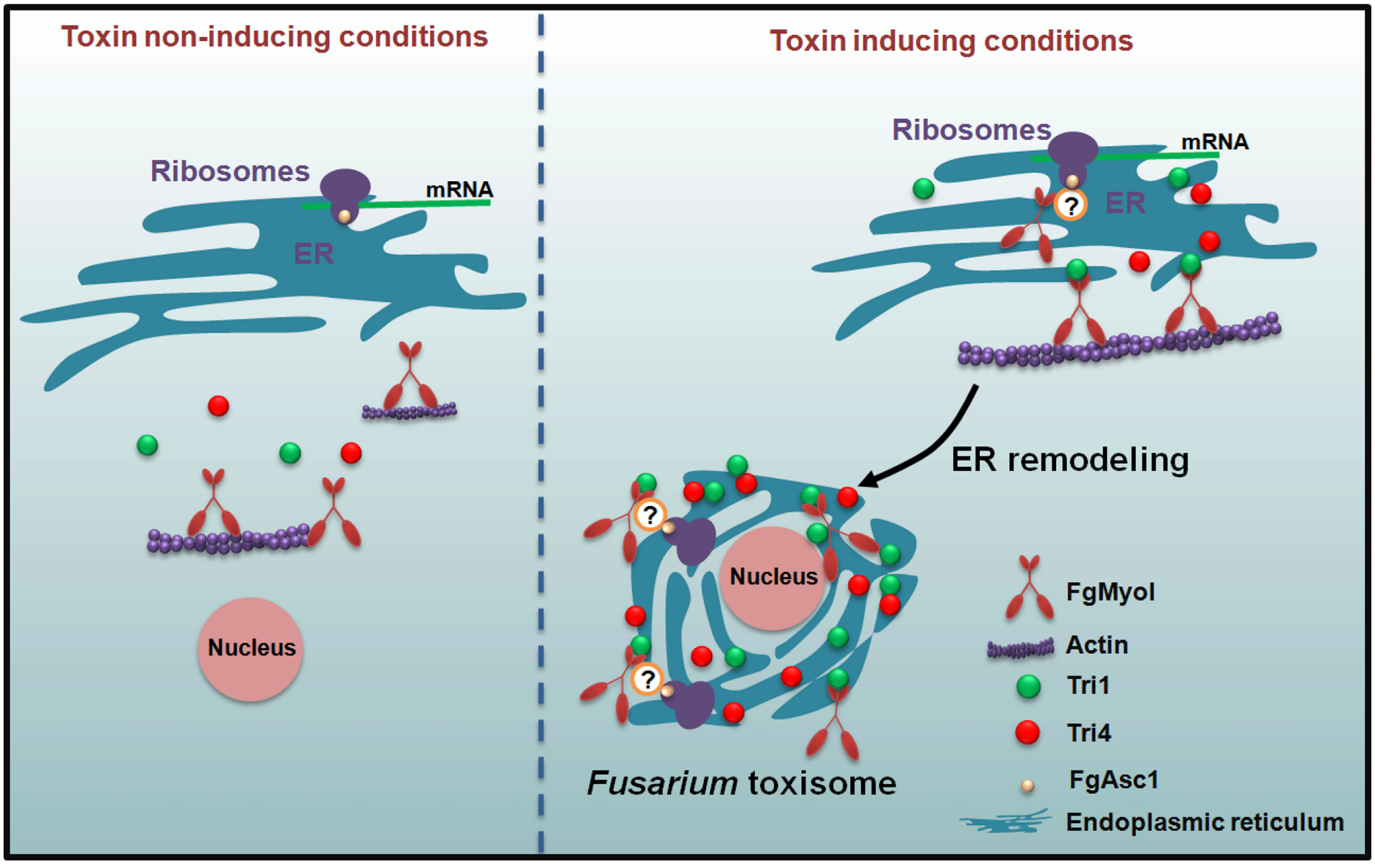
A proposed model showing the role of FgMyo1 in toxisome formation. Trichothecene biosynthesis enzymes (Tri proteins) are produced at a low level under toxin noninducing conditions. In toxin inducing conditions, FgMyo1 directly participates in remodeling the endoplasmic reticulum (ER) via the myosin-actin cytoskeleton to form the spherical and ovoid structures termed “*Fusarium* toxisomes.” In addition, FgMyo1 interacts with FgAsc1 indirectly to enhance the translation of Tri proteins. Phenamacril is able to suppress toxisome formation by inhibiting the ATPase activity of FgMyo1, and subsequently reduces the biosynthesis of DON in *Fusarium graminearum*.

The myosin-actin system also involves in the movement of organelles within cells, including the organelles for secondary metabolites organization. For instance, the short transportation of melanosomes for the skin pigment melanin biosynthesis at the peripheral region of the mammalian cell is largely dependent on the Rab27a, melanophilin, myosinV-actin filament complex [62]. In *Fusarium* spp. Tri12 is suggested to play a role in export of trichothecene mycotoxins, which forms vacuoles and vesicles during the mycotoxin inducing condition [20, 21]. A previous study suggested that Tri12 interacted with toxisomes and may transfer the trichothecenes from toxisomes into the vesicles and vacuoles for further export [23]. The motility of vesicles containing Tri12 was reversibly inhibited by latrunculin A, indicating that movement was dependent upon the filamentous actin [21, 23]. The motor proteins are needed for the cellular motility of Tri12 by mechanical driving force on the filamentous actin. There are three major super-families of motor proteins: kinesins, dyneins, and myosins. The first two act as motors on microtubule filaments, while myosins function on actin [63]. Thus, it would be interesting to further study the functions of myosins in the transport of toxins that may accumulate in Tri12-linked vacuoles and vesicles in *F*. *graminearum* and in other toxigenic fungi. Taken together, our data support a model in which FgMyo1 is essential for toxisome formation under the DON induction conditions in *F*. *graminearum* by interacting with FgAsc1 indirectly for regulating the Tri protein biosynthesis and by directly participating in the endoplasmic reticulum (ER) remodeling via the myosin-actin cytoskeleton system. In addition, the small molecule phenamacril is able to suppress the toxisome formation by inhibiting the ATPase activity of FgMyo1 (Fig 7).

## Material and methods

### Fungal strains and growth assays

The *F*. *graminearum* wild-type strain PH-1 (NRRL 31084) was used as a parental strain. The wild-type strain and transformants generated in this study were grown on potato dextrose agar (PDA) and minimal medium (MM) for hyphal examination. The carboxymethyl cellulose (CMC) liquid medium was used for conidiation assays [64]. For toxisome observation and trichothecene production analysis, each strain was grown in liquid trichothecene biosynthesis inducing (TBI) medium [38] at 28 °C in a shaker (150 rpm) in the dark. Each experiment was repeated three times.

### Strain construction

The strains ΔFgPrk1, ΔFgEnd3, ΔFgTri1, ΔFgTri4, ΔFgAsc1, ΔFgAurJ and ΔFgAurF were constructed using the protocol described previously [65]. Briefly, the open reading frame (ORF) of each gene was replaced with hygromycin resistance cassette (*HPH*) and subsequent deletion mutants were identified by PCR assays with relevant primers (S2 Table). For complementation, each ORF fused with a tag and geneticin resistance gene was introduced into corresponding mutant, and transformants were selected with geneticin. To construct FgMyo1 silenced mutants, a 540 bp fragment was amplified and inserted forward and reverse into the pSilent-1 plasmid, and the recombination hairpin RNA silencing plasmid was introduced into PH-1 as previous described [25]. To replace the *FgMYO1* promoter with P_zear_, the *HPH* and P_zear_ fragments were amplified respectively and fused by overlap PCR. Subsequently, the “HPH-P_zear_” fragment was further fused with the 5′ and 3′ flanking regions of the *FgMYO*1 gene. The resulting fusion fragment was purified and transformed into PH-1. To induce the P_zear_ replacement, the inducer β-estradiol at 30 μM was added to the medium during the regeneration and mutant selection processes [66].

To construct the FgTri1-GFP fusion cassette, the FgTri1 fragment containing the native promoter and ORF (without stop codon) was amplified with primers A15 + A16 (S1 Table). The resulting PCR products were co-transformed with Xho1-digested pYF11 into XK1-25. The alkali-cation yeast transformation kit (MP Biomedicals, Solon, USA) was used to generate the recombined FgTri1-GFP fusion vector. Subsequently, the FgTri1-GFP fusion vector was recovered from the yeast transformant by using the yeast plasmid extract kit (Solarbio, Beijing, China) and then transferred into *E*.*coli* strain DH5α for amplification. Using the same strategy, other GFP or RFP fusion cassettes were also constructed. Each recombination plasmid was transformed into PH-1 or the corresponding mutant for generating fluorescent label strains.

### Screening for toxisome formation inhibitors

The strain expressing the FgTri1-GFP in the ΔTri1 background was used as the fluorescent reporter strain for anti-toxisome formation screening. The TBI medium supplemented with 10^4^ conidia/mL was added into a 24-well plate (2.0 mL/well). After 24 h static incubation at 28 °C, each tested compound was added into a well and the plate was incubated for another 48 h. Then, the fluorescent intensity in each well was scanned with the Varioskan Flash Multimode Reader (Thermo Scientific, MA, USA) for first round screening. The wells with lower or no fluorescent signals compared with that of the control treatment (the same volume of solvent dimethyl sulfoxide, DMSO) were further observed by a confocal microscopy. A total of 131 antifungal compounds including 11 commercialized fungicides were tested for the activity against toxisome formation. For each compound, there were three-well replicates, and the experiment was repeated three times.

### Microscopic examinations

The fluorescent intensity and localization of tagged proteins were observed with a Zeiss LSM780 confocal microscopy (Gottingen, Niedersachsen, Germany). For observation of toxisome formation patterns in PH-1 and derived mutants, each strain labeled with FgTri1-GFP was cultured in TBI for 48 h before examination. All samples were mounted on glass slides and sealed with cover glasses. The following parameter sets of the confocal microscopy were used: Plan-Neofluar 40x/1.30 Oil DIC M27 objective; laser: at 488 nm at 50% power for green fluorescence; dimension of X = 70.78 μm, Y = 70.78 μm; pinhole: 90 μm; digital gain: 1.00. To observe toxisomes *in planta*, fresh mycelial plugs of the fluorescent reporter strain were inoculated on the leaves of wheat seedlings of a susceptible cultivar Jimai 22. After incubation at 25°C and 100% RH (relative humidity) for 5 days, the infected leaves were taken for toxisome examination observed under Plan-Neofluar 20x/0.50 M27 objective.

The following filter sets were used for other fluorescent or dye staining: the laser excitation wavelength was set at 405 nm for DAPI (blue fluorescence), at 561 nm for FM4-64 or RFP/mCherry (red fluorescence), at 514 nm for YFP (yellow fluorescence). The endoplasmic reticulum (ER) was stained with ER-Tracker Red (Beyotime technology Co., Ltd), and laser was set at 587 nm for red fluorescence. The intensity of fluorescence was acquired using the Zeiss ZEN 2010 software.

### Bimolecular Fluorescence Complementation (BiFC) assays

For BiFC assays, the final plasmid constructs of pYFPN-FgTri1 and pFgMyo1-YFPC were verified by sequencing and then co-transformed into the protoplasts of PH-1 in pairs. Transformants resistant to both hygromycin and neomycin were isolated and confirmed by PCR. The recombination plasmid pYFPN-FgTri1 or pFgMyo1-YFPC was transformed into PH-1, and resulted transformants were used as negative controls. YFP signals in the mycelia grown in TBI for 48 h were examined under a Zeiss LSM780 confocal microscope (Gottingen, Niedersachsen, Germany).

### Western blotting hybridization

The protein isolation was performed as described previously [67]. The resulting proteins were separated by 10% sodium dodecyl sulfate-polyacrylamide gel electrophoresis (SDS-PAGE) and transferred to Immobilon-P transfer membrane (Millipore, Billerica, MA, USA). The polyclonal anti-Flag A9044 (Sigma, St. Louis, MO) and monoclonal anti-GFP ab32146 (Abcam, Cambridge, UK) antibodies were used at a 1:5000 to 1:10 000 dilution for immunoblot analyses. The samples were also detected with monoclonal anti-GAPDH antibody EM1101 (Hangzhou HuaAn Biotechnology Co., Ltd.) as a reference. The intensity of immunoblot bands were quantified using the ImageQuantTL software.

### Analysis of mycotoxin production

To quantify the mycotoxin production, each strain was grown in TBI medium or inoculated on wheat kernels. DON was extracted, and then purified, and quantified using the LC-MS/MS system as described previously [5, 68].

### Affinity capture-mass spectrometry analysis

The bait protein FgMyo1 was dual labeled with ZZ tag and 3×Flag at its N-terminus and C-terminus, respectively. The resulting fusion cassette was transferred into PH-1. The resulting transformant (PH-1::ZZ-FgMyo1-3×Flag) was used for protein extraction as previous described previously [65] and the affinity capture was conducted by the following procedures. After protein extraction, supernatant (25 ml) was transferred into a sterilized tube. The first run affinity capture was conducted using rabbit IgG agarose beads (Haoran Biotech Co., Shanghai, China), which was immuno-interacted with the ZZ tag. A total of 500 μl IgG agarose beads were added into the above supernatant to capture ZZ-FgMyo1-3×Flag interacting proteins, following the manufacturer’s instructions (General Electric Company, GA, USA). Then, the washed beads were subjected for the second run capture with anti-Flag agarose beads according to the manufacturer’s instructions (Abmart, NJ, USA). The final ZZ-FgMyo1-3×Flag interacting proteins captured by the anti-Flag agarose beads were eluted with TBS supplemented with 10% SDS. In addition, the ZZ-FgTri1-3×Flag was constructed and the interacting proteins were captured using the same strategy. The captured proteins were digested with trypsin and further analyzed by mass spectrometry using a previous published protocol [69]. Enrichment for proteins assigned to particular functional categories (FunCat) was calculated as described previously [20, 28].

### Co-immunoprecipitation (Co-IP) assays

The GFP, RFP, 3× Flag, or mCherry-fusion constructs were verified by DNA sequencing and transformed in pairs into PH-1. Transformants expressing pairs of fusion constructs were confirmed by western blot analysis. In addition, the transformants expressing a single fusion construct were used as references. For Co-IP assays, total proteins were extracted and incubated with the anti-GFP (ChromoTek, Martinsried, Germany) or anti-Flag (Abmart, Shanghai, China) agarose as described above. Proteins eluted from agarose were analyzed by western blot detection with a polyclonal anti-Flag A9044 (Sigma, St. Louis, MO), or an anit-GFP antibody (Abcam, Cambridge, UK). The protein samples were also detected with monoclonal anti-GAPDH antibody EM1101 (Hangzhou Huaan Biotechnology Co., Ltd.) as a reference. Each experiment was repeated twice.

## Supporting information

S1 Fig Chemical structure of the novel antifungl compound phenamacril. (TIF)

S2 Fig Time course analysis of accumulation and localization of Tri1-GFP in the toxin inducing conditions. **(A)** Examination for toxisome formation in time. The images were taken after the strain ΔTri1::Tri1-GFP was incubated in TBI at the corresponding time indicated in the figure. Bar = 10 μm. **(B)** The abundance of Tri1-GFP protein at the corresponding time was determined by the western blot assay with the anti-GFP antibody. The protein samples were also incubated with the anti-GAPDH antibody as a reference. **(C)** Time course analysis of production of DON by ΔTri1::Tri1-GFP in TBI.
(TIF)

S3 Fig Tri4-RFP was co-localized with Tri1-GFP at toxisomes in hyphae of PH-1::Tri1-GFP+Tri4-RFP grown in the toxin inducing medium TBI. DIC indicates differential interference contrast. Bar = 10 μm.
(TIF)

S4 Fig Inhibition of antifungal compounds against toxisome formation. **(A)** The inhibition of each compound (at 0.5 μg/ml) against mycelial growth of *F*. *graminearum* on PDA. The solvent DMSO was used as a control. **(B**) Toxisome formation in the mycelia of ΔTri1::Tri1-GFP treated with each antifungal compound. After the strain was cultured in TBI for 24 h, each fungicide was added into TBI at the final concentration at 0.5 μg/ml. Subsequently, the strain was incubated for another 24 h before observation. The DMSO is the solvent control. **(C**) Production of DON in each treatment. DON was extracted from mycelia of each strain cultured in TBI for 7 days. Values on the bars followed by different letters are significantly different according to a Fisher’s least significant difference (LSD) test at *P* = 0.05.
(TIF)

S5 Fig Examination for toxisome formation in hyphae of ΔTri1::Tri1-GFP treated with 0.5 μg/ml phenamacril for different times. **(A)** Toxisome formation patterns in ΔTri1::Tri1-GFP grown in TBI for the times as indicated in the figure. Bar = 10 μm. **(B)** Phenamacril abolished the toxisome formation in ΔTri1::Tri1-GFP. After ΔTri1::Tri1-GFP was grown in TBI for 24 h, the culture was then treated with phenamacril for the additional time (from 6 to 48 h) as indicated in the figure. Bar = 10.
(TIF)

S6 Fig FgMyo1 derived mutants and the actin associated protein gene deletion mutants ΔFgPrk1 and ΔFgEnd3 attenuated virulence on flowering wheat heads. Infected wheat heads were examined 15 days after inoculation with conidial suspension of each strain. The inoculation sites were indicated as black dots.
(TIF)

S7 Fig Comparisons in localization of the ribosomal 60S subunit protein L25 (FgRpL25 tagged with mCherry) in toxin non-inducing (upper panel) and toxin inducing conditions (lower panel). The strain was also stained with a nucleus tracker DAPI (4′, 6-diamidino-2-phenylindole). Bar = 10 μm.
(TIF)

S8 Fig Thin reticulate ER patterns in mycelia of *F*. *graminearum* grown in the non-toxin inducing medium. The mycelia of PH-1 grown in PDB for 48 h were used for staining with the ER-tracker Red. Bar = 10 μm.
(TIF)

S9 Fig Schematic structures of FgMyo1 and the pleckstrin homology motif. **(A)** Schematic structures of the FgMyo1 protein in *F*. *graminearum*. **(B)** Alignment of the FgMyo1 pleckstrin homology (PH) domain (residues 814–837 aa) with its orthologs of *Homo sapiens*. Red residues indicate the conserved basic residues that are important for membrane binding in PH domain. Accession numbers for the proteins listed are indicated.
(TIF)

S10 Fig The myosin I mediated endocytosis process is not connected with the toxisome formation in *F*. *graminearum*. **(A)** Time-course of FM4-64 internalization via the endocytic pathway in the wild type, FgMyo1^E420K^, ΔFgPrk1 and ΔFgEnd3. Living cells grown in PDB were stained with 8 mM FM4-64. Bar = 10 mm. **(B)** Hyphal growth patterns of endocytosis mutant ΔFgAPM4 and ΔFgAbp1 on PDA. **(C)** Toxisome formation of ΔFgAPM4 and ΔFgAbp1 grown in TBI medium. **(D)** The DON production of ΔFgAPM4, ΔFgAbp1 and their complemented strains. The DON was extracted from mycelia of each strain grown in TBI for 7 days. Values on the bars followed by the same letter are not significantly different according to a Fisher’s least significant difference (LSD) test at *P* = 0.05.
(TIF)

S1 Table Identification of Tri1 and FgMyo1-interacting proteins by the affinity capture-mass spectrometry assay. (DOCX)

S2 Table A list of primers used in this study. (DOCX)
