# Supplementary figures and images for: The fungal myosin I is essential for Fusarium toxisome formation
Source: PLoS Pathog. 2018 Jan 22;14(1):e1006827. doi: 10.1371/journal.ppat.1006827 (PMC5794197; doi:10.1371/journal.ppat.1006827)

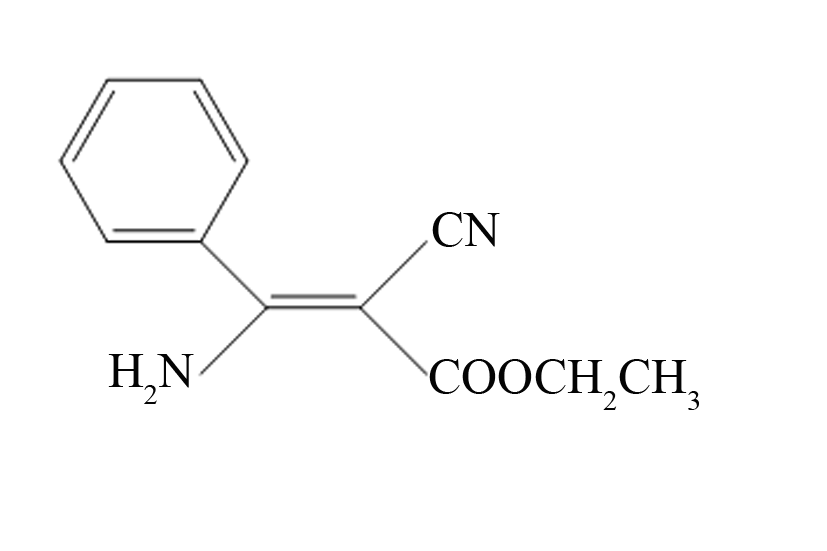

Supplement: S1 Fig — (TIF) [file ppat.1006827.s001.tif]

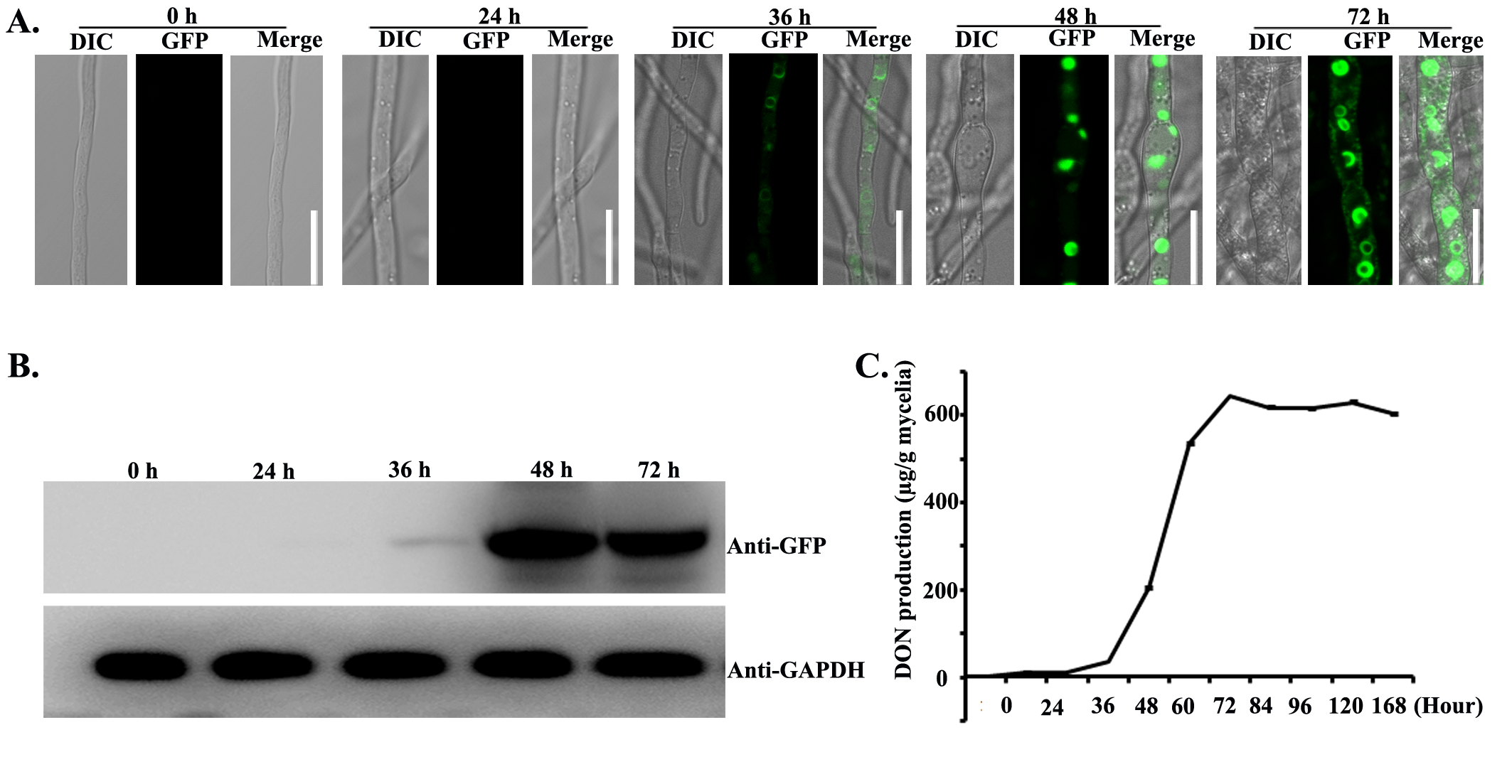

Supplement: S2 Fig — (A) Examination for toxisome formation in time. The images were taken after the strain ΔTri1::Tri1-GFP was incubated in TBI at the corresponding time indicated in the figure. Bar = 10 μm. (B) The abundance of Tri1-GFP protein at the corresponding time was determined by the western blot assay with the anti-GFP antibody. The protein samples were also incubated with the anti-GAPDH antibody as a reference. (C) Time course analysis of production of DON by ΔTri1::Tri1-GFP in TBI. (TIF) [file ppat.1006827.s002.tif]

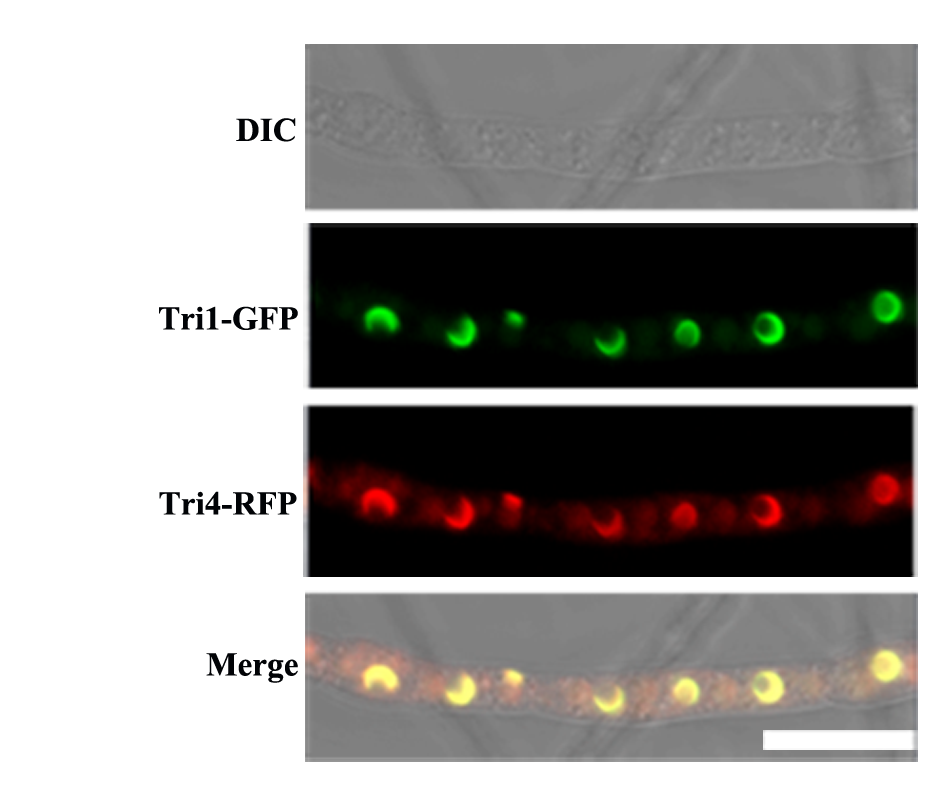

Supplement: S3 Fig — DIC indicates differential interference contrast. Bar = 10 μm. (TIF) [file ppat.1006827.s003.tif]

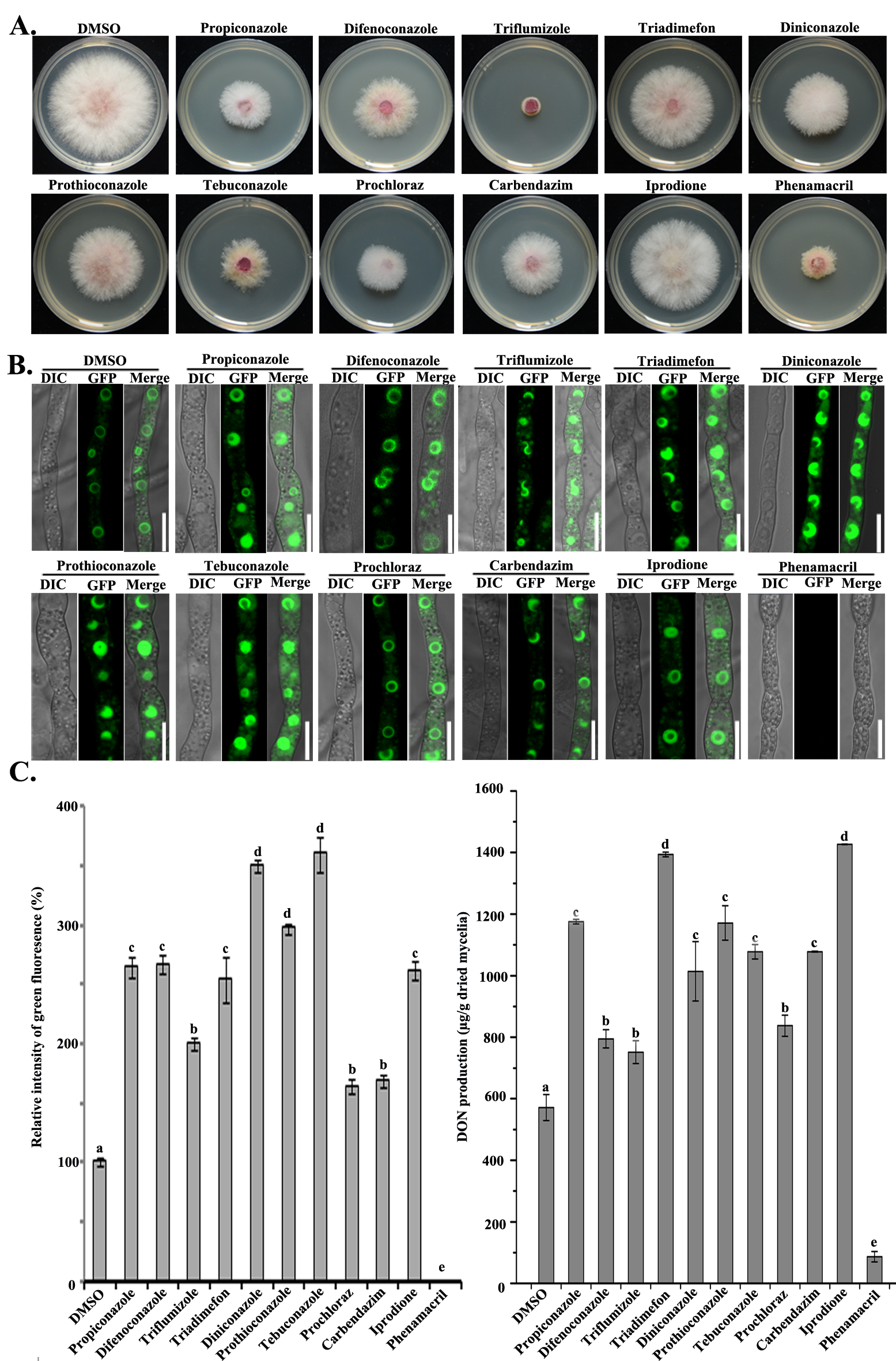

Supplement: S4 Fig — (A) The inhibition of each compound (at 0.5 μg/ml) against mycelial growth of F. graminearum on PDA. The solvent DMSO was used as a control. (B) Toxisome formation in the mycelia of ΔTri1::Tri1-GFP treated with each antifungal compound. After the strain was cultured in TBI for 24 h, each fungicide was added into TBI at the final concentration at 0.5 μg/ml. Subsequently, the strain was incubated for another 24 h before observation. The DMSO is the solvent control. (C) Production of DON in each treatment. DON was extracted from mycelia of each strain cultured in TBI for 7 days. Values on the bars followed by different letters are significantly different according to a Fisher’s least significant difference (LSD) test at P = 0.05. (TIF) [file ppat.1006827.s004.tif]

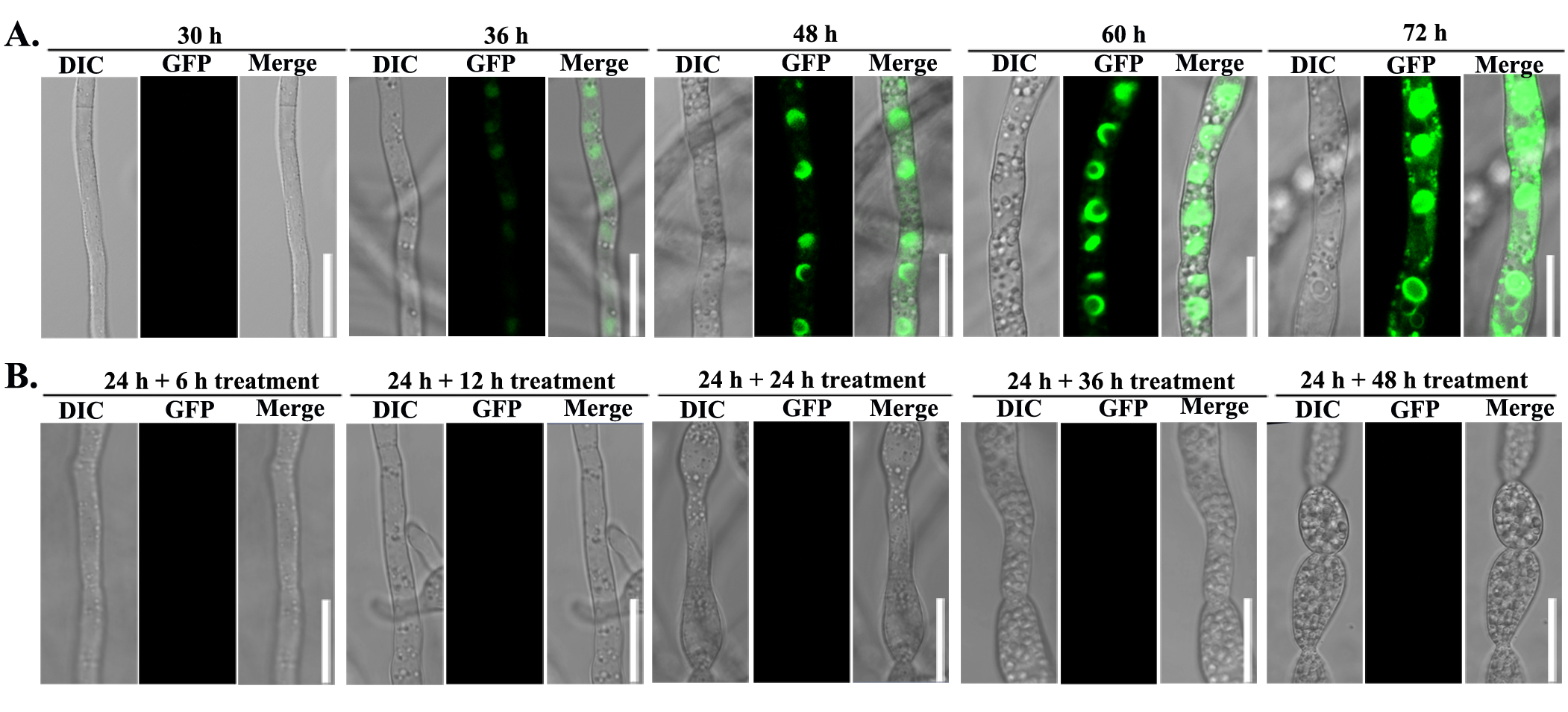

Supplement: S5 Fig — (A) Toxisome formation patterns in ΔTri1::Tri1-GFP grown in TBI for the times as indicated in the figure. Bar = 10 μm. (B) Phenamacril abolished the toxisome formation in ΔTri1::Tri1-GFP. After ΔTri1::Tri1-GFP was grown in TBI for 24 h, the culture was then treated with phenamacril for the additional time (from 6 to 48 h) as indicated in the figure. Bar = 10. (TIF) [file ppat.1006827.s005.tif]

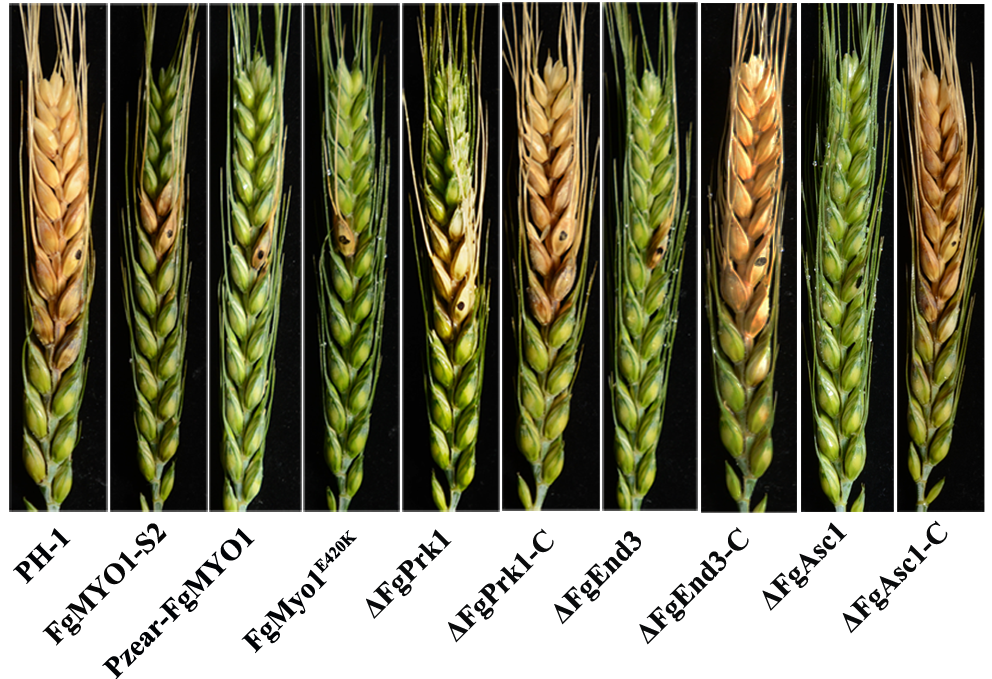

Supplement: S6 Fig — Infected wheat heads were examined 15 days after inoculation with conidial suspension of each strain. The inoculation sites were indicated as black dots. (TIF) [file ppat.1006827.s006.tif]

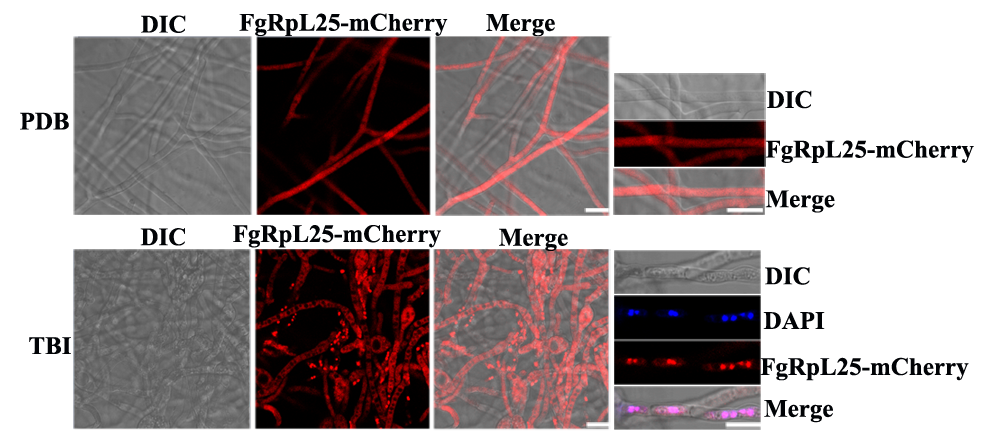

Supplement: S7 Fig — The strain was also stained with a nucleus tracker DAPI (4′, 6-diamidino-2-phenylindole). Bar = 10 μm. (TIF) [file ppat.1006827.s007.tif]

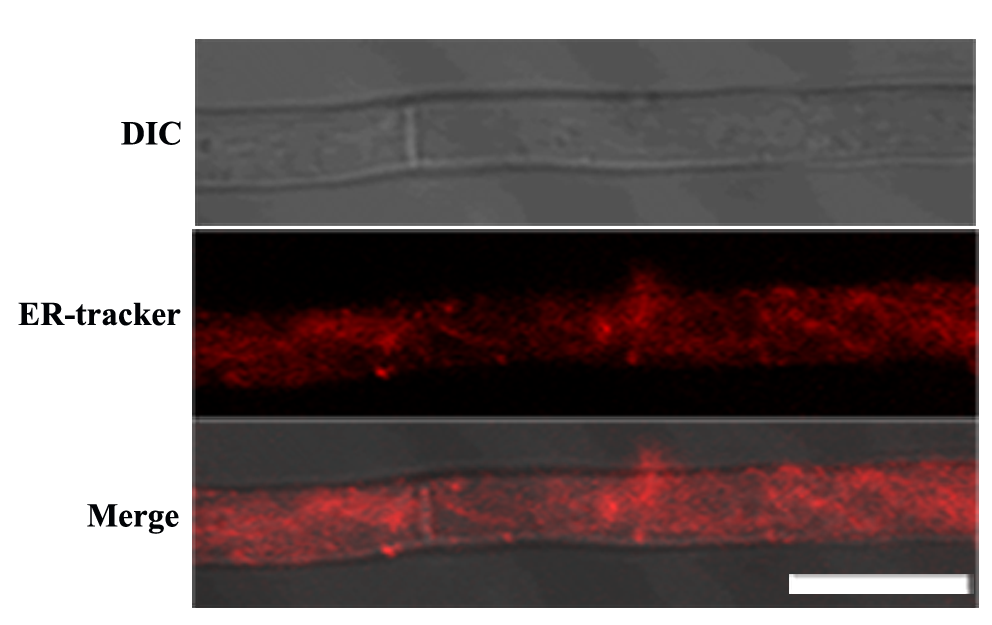

Supplement: S8 Fig — The mycelia of PH-1 grown in PDB for 48 h were used for staining with the ER-tracker Red. Bar = 10 μm. (TIF) [file ppat.1006827.s008.tif]

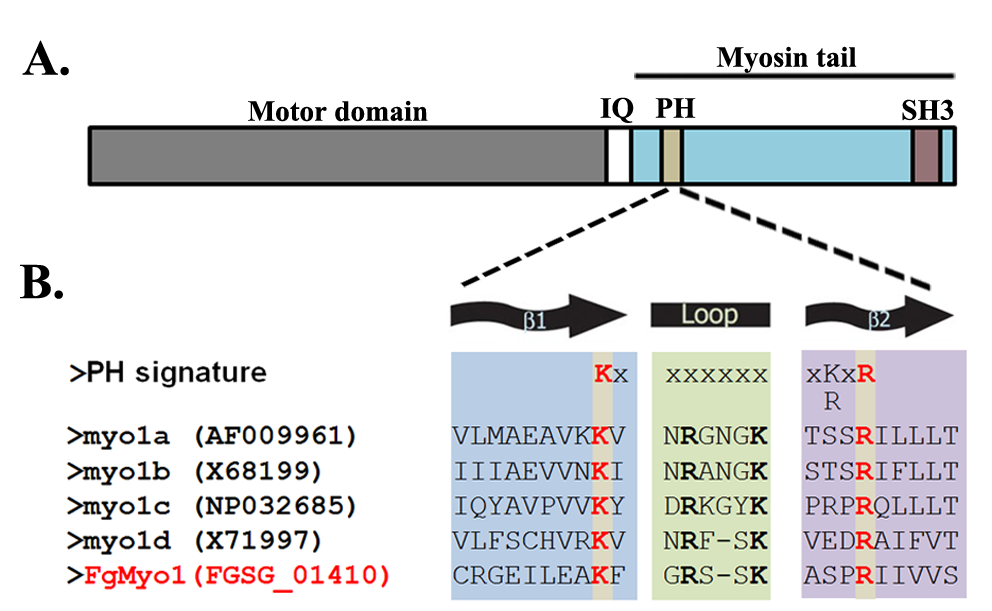

Supplement: S9 Fig — (A) Schematic structures of the FgMyo1 protein in F. graminearum. (B) Alignment of the FgMyo1 pleckstrin homology (PH) domain (residues 814–837 aa) with its orthologs of Homo sapiens. Red residues indicate the conserved basic residues that are important for membrane binding in PH domain. Accession numbers for the proteins listed are indicated. (TIF) [file ppat.1006827.s009.tif]

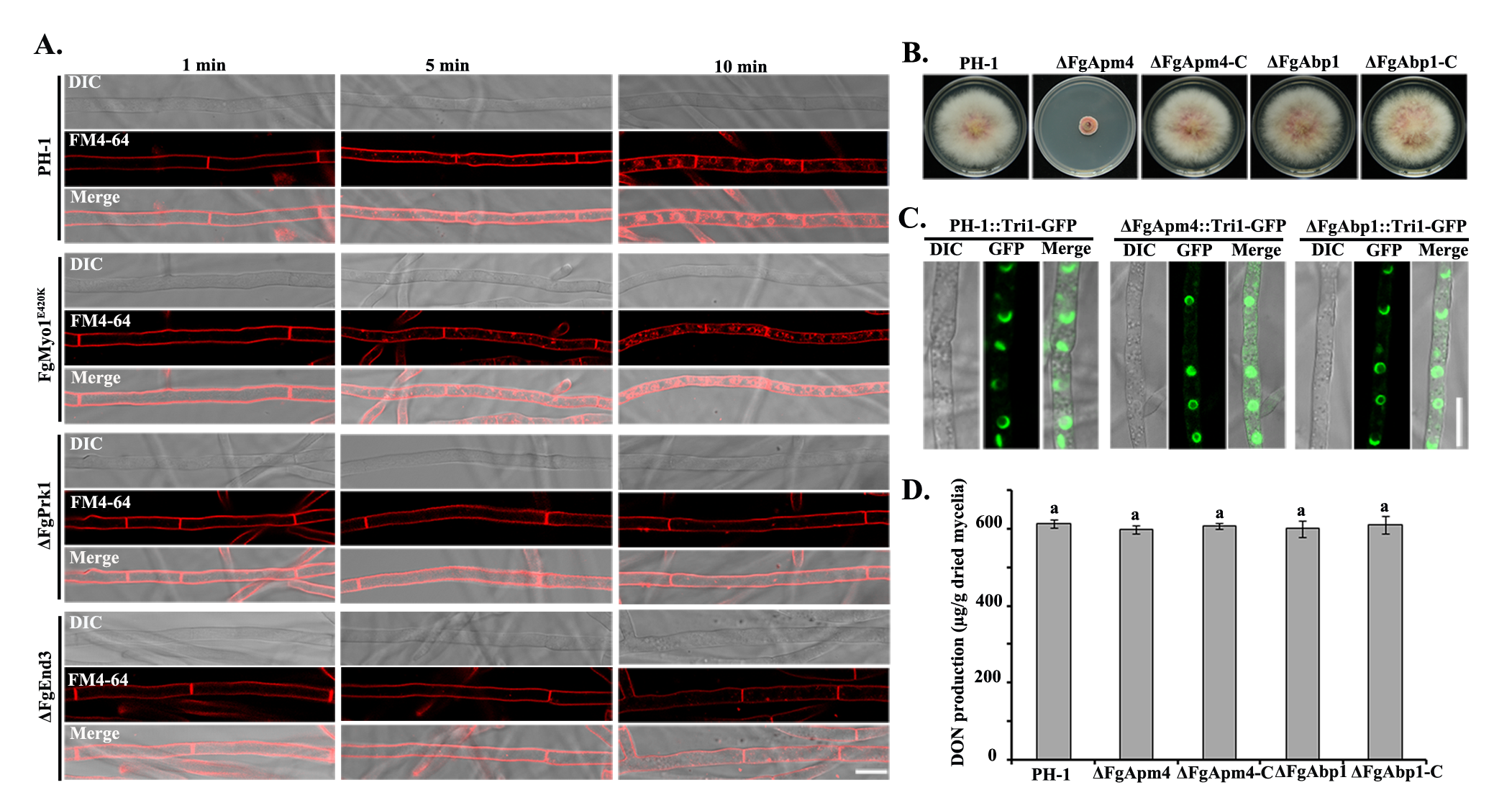

Supplement: S10 Fig — (A) Time-course of FM4-64 internalization via the endocytic pathway in the wild type, FgMyo1E420K, ΔFgPrk1 and ΔFgEnd3. Living cells grown in PDB were stained with 8 mM FM4-64. Bar = 10 mm. (B) Hyphal growth patterns of endocytosis mutant ΔFgAPM4 and ΔFgAbp1 on PDA. (C) Toxisome formation of ΔFgAPM4 and ΔFgAbp1 grown in TBI medium. (D) The DON production of ΔFgAPM4, ΔFgAbp1 and their complemented strains. The DON was extracted from mycelia of each strain grown in TBI for 7 days. Values on the bars followed by the same letter are not significantly different according to a Fisher’s least significant difference (LSD) test at P = 0.05. (TIF) [file ppat.1006827.s010.tif]
