# Supplementary material for: The fungal myosin I is essential for Fusarium toxisome formation
Source: PLoS Pathog. 2018 Jan 22;14(1):e1006827. doi: 10.1371/journal.ppat.1006827 (PMC5794197; doi:10.1371/journal.ppat.1006827)
Supplement: S1 Table — (DOCX) [file ppat.1006827.s011.docx]

**Table S1. Identification of Tri1 and FgMyo1-interacting proteins by the affinity capture-mass spectrometry assay**

| Protein ID | Description | Orthologs in *S. cerevisiae* |
| --- | --- | --- |
| ZZ-Tri1-Flag |  |  |
| FGSG_00071 | Tri1 calonectrin oxygenase | - |
| FGSG_10855 | Alcohol dehydrogenase 1 | TEF1 |
| FGSG_08811 | Elongation factor 1-alpha | ADH3 |
| FGSG_07335 | Actin | ACT1 |
| FGSG_09321 | Acetyl-CoA acetyltransferase IB | ERG10 |
| FGSG_03535 | trichodiene oxygenase-Tri4 | - |
| FGSG_01410 | FgMyo1 | MYO5 |
| FGSG_01346 | Enolase | ENO2 |
| FGSG_00838 | Heat shock protein Hsp70 | SSA4 |
| FGSG_09870 | Guanine nucleotide-binding protein subunit beta-like protein | ACS1 |
| FGSG_09471 | Glucose-regulated protein | KAR2 |
| FGSG_08737 | Woronin body major protein | - |
| FGSG_10873 | GTP-binding protein Ypt1 | YPT1 |
| FGSG_06646 | GTP-binding protein Sar1 | SAR1 |
| FGSG_09016 | Protein transport protein SEC61 subunit alpha | SEC61 |
| FGSG_06245 | Cofilin/tropomyosin-type actin-binding protein | COF1 |
| FGSG_08621 | F-actin capping protein subunit alpha | CAP1 |
| FGSG_03540 | Tri11 isotrichodermin C-15 hydroxylase | - |
| FGSG_06878 | Calcium/calmodulin-dependent protein kinase | CMK2 |
| FGSG_06209 | GTP-binding protein Sas1 | SAS1 |
| FGSG_09271 | Protein transport protein SEC13 | SEC13 |
| FGSG_01956 | Ubiquitin-2 like Rad60 SUMO-like | RPL40B |
| FGSG_06392 | Profilin | PFY1 |
| FGSG_10856 | Actin-like protein 3 | ARP3 |
| FGSG_10180 | Coatomer epsilon subunit | - |
| FGSG_06611 | Tubulin beta chain | TUB2 |
| FGSG_04378 | C2 domain | - |
| FGSG_04485 | Actin-like protein 2 | APR2 |
| FGSG_09197 | HMR1 HMG-CoA reductase | HMG2 |
| FGSG_01226 | F-actin capping protein subunit beta | CAP2 |
|  |  |  |
| ZZ-FgMyo1-Flag |  |  |
| FGSG_01410 | FgMyo1 | MYO5 |
| FGSG_03992 | Phosphoglycerate kinase | - |
| FGSG_01425 | Plasma membrane ATPase | PMA1 |
| FGSG_01049 | Hypothetical protein | - |
| FGSG_00071 | Cytochrome P450 monooxygenase -Tri1 | - |
| FGSG_05088 | kinesin heavy chain | KAR3 |
| FGSG_01956 | Ubiquitin-2 like Rad60 SUMO-like | RPL40B |
| FGSG_07335 | Actin | ACT1 |
| FGSG_05586 | Protein Kinase Prk1 | PRK1 |
| FGSG_02105 | AP-2 complex subunit mu-1 | APM1 |
| FGSG_07571 | Hypothetical protein | - |
| FGSG_01316 | Abp1, actin binding protein | ABP1 |
| FGSG_00252 | Hypothetical protein | - |
| FGSG_09870 | Guanine nucleotide-binding protein subunit beta-like protein | ASC1 |
| FGSG_03498 | Cytochrome P450 | - |
| FGSG_01891 | Calmodulin | CMD1 |
| FGSG_05306 | Vps15 protein kinase | VPS15 |
| FGSG_01226 | F-actin capping protein subunit beta | CAP2 |
| FGSG_12975 | Cell division cycle protein CDC37 | CDC37 |
| FGSG_10080 | Delta-aminolevulinic acid dehydratase | HEM2 |
| FGSG_10287 | Penicillin-binding protein | - |
| FGSG_08621 | F-actin capping protein subunit alpha | CAP2 |
| FGSG_09197 | HMR1 HMG-CoA reductase (HMG2/HMG1; HMGCR) | HMG2 |
| FGSG_01445 | 6-phosphofructo-2-kinase | - |
| FGSG_01783 | Terpene synthase family, metal binding domain | - |
| FGSG_00499 | Ribophorin I | - |
| FGSG_02479 | GTP-binding protein Ypt6 | YPT6 |
| FGSG_11894 | ATP synthase subunit alpha | - |
| FGSG_03933 | Putative cyclase | - |
| FGSG_08897 | Pre-mRNA-splicing factor Slu7 | SLU7 |
