# Supplementary material for: The fungal myosin I is essential for Fusarium toxisome formation
Source: PLoS Pathog. 2018 Jan 22;14(1):e1006827. doi: 10.1371/journal.ppat.1006827 (PMC5794197; doi:10.1371/journal.ppat.1006827)
Supplement: S2 Table — (DOCX) [file ppat.1006827.s012.docx]

**Table S2. A list of primers used in this study**

| Name | Sequence (5'to 3') | Products |
| --- | --- | --- |
| FGSG_00071-P1 | TTGTGAGTAGGCCTCATA | Upstream fragment of *FgTri1* |
| FGSG_00071-P2 | CAAAATAGGCATTGATGTGTTGACCTCCGACAGCGAAATGGTCTGTC |  |
| FGSG_00071-P3 | CTCGTCCGAGGGCAAAGGAATAGAGTAGGTAGGAGGACGTCACAGTCTTG | Downstream fragment of *FgTri1* |
| FGSG_00071-P4 | GCCTCAGTGACCGAGGTAG |  |
| FGSG_00071-ID-F | CTTATACTTAGGACCCTACCGAT | Identification *FgTri1* deletion transformants |
| FGSG_00071-ID-R | TGGCGAGATCAAACCCAAC |  |
| FGSG_03535-P1 | GGCTTGAGCCTTGATGGGATG | Upstream fragment of *FgTri4* |
| FGSG_03535-P2 | CAAAATAGGCATTGATGTGTTGACCTCCCTTCTAAAGCTCAAGTCTTTG |  |
| FGSG_03535-P3 | CTCGTCCGAGGGCAAAGGAATAGAGTAGGGCGCATCTGACAAACTGTC | Downstream fragment of *FgTri4* |
| FGSG_03535-P4 | GACTGGACAGTTGGAGAGAT |  |
| FGSG_03535-ID-F | ACAACGCATGCAAAGCACTCT | Identification *FgTri4* deletion transformants |
| FGSG_03535-ID-R | ATTAGTAATGCAGAGAAGACT |  |
| FGSG_02326-P1 | GACTTGACTTACTTCTTGAACG | Upstream fragment of *FgAurJ* |
| FGSG_02326-P2 | CAAAATAGGCATTGATGTGTTGACCTCCTGTGGCTAGGCTGGCTAGTGA |  |
| FGSG_02326-P3 | CTCGTCCGAGGGCAAAGGAATAGAGTAGTATTATCTATTTGGGGGAGGC | Downstream fragment of *FgAurJ* |
| FGSG_02326-P4 | GACTGGACAGTTGGAGAGAT |  |
| FGSG_02326-ID-F | GTTACGGAGCCTAGTCCGAA | Identification *FgAurJ* deletion transformants |
| FGSG_02326-ID-R | AACGTGATTGGACTGACCAT |  |
| FGSG_02327-P1 | GTCTTCTTTACACTTCCTGGC | Upstream fragment of *FgAurF* |
| FGSG_02327-P2 | CAAAATAGGCATTGATGTGTTGACCTCCTGTCAGTGAAACAAAACGACA |  |
| FGSG_02327-P3 | CTCGTCCGAGGGCAAAGGAATAGAGTAGGATGTTTGTTACTTTGATATTT | Downstream fragment of *FgAurF* |
| FGSG_02327-P4 | TATGGACCGCTTCTGTATG |  |
| Fg02327-ID-F | GTTCGGACTAGGCTCCGT | Identification *FgAurF*  deletion transformants |
| Fg02327-ID-R | GATACAATCAACAACATTGCA |  |
| HPH-F | GGAGGTCAACACATCAATGCCTATT | Fragment of HPH (Hygromycin B) resistance gene |
| HPH-R | CTACTCTATTCCTTTGCCCT |  |
| NEO-F | GGAGGTCAACACATCAATGCT | Fragment of Neo (Neomycin) resistance gene |
| NEO-R | TCAGAAGAACTCGTCAAGAAG |  |
| FGSG_05586-P1 | GCCGAGTATAGATTCACAACT | Upstream fragment of *FgPrk1* |
| FGSG_05586-P2 | CAAAATAGGCATTGATGTGTTGACCTCCCGTGGGCGGAAGTGTCGAGAC |  |
| FGSG_05586-P3 | CTCGTCCGAGGGCAAAGGAATAGAGTAGATAGACAATGGTGAAGGTATC | Downstream fragment of *FgPrk1* |
| FGSG_05586-P4 | CGATAGGTGCCTTGATGTTG |  |
| FGSG_05586-ID-F | AACTCCAGTATACCCACCTGG | Identification *FgPrk1*  deletion transformants |
| FGSG_05586-ID-R | ACAGGCTTTTCCTTGAGAACC |  |
| FGSG_09721-P1 | GTGGTAGTTGTTGTAGACAT | Upstream fragment of *Fg**End3* |
| FGSG_09721-P2 | CAAAATAGGCATTGATGTGTTGACCTCCATTACGTTTGTGGGCTCTGG |  |
| FGSG_09721-P3 | CTCGTCCGAGGGCAAAGGAATAGAGTAGGAAGAGGTTCGCTTAAAGCG | Downstream fragment of *Fg End3* |
| FGSG_09721-P4 | CCAGCCTTCATCGTACTGCCG |  |
| FGSG_09721-ID-F | ACATCACATATTCTTGATTC | Identification *Fg End3*  deletion transformants |
| FGSG_09721-ID-R | CCCACTCCGCAGTCTAATTA |  |
| FGSG_09870-P1 | GATGCTCTCAGACGACTGGT | Upstream fragment of *FgASC1* |
| FGSG_09870-P2 | CAAAATAGGCATTGATGTGTTGACCTCCTGCGGTGACGAGGCGATGCAGAG |  |
| FGSG_09870-P3 | CTCGTCCGAGGGCAAAGGAATAGAGTAGTATACACATGTCTAGATTCA | Downstream fragment of *FgASC1* |
| FGSG_09870-P4 | CGATTGGAAACGCGTGAATAAC |  |
| FGSG_09870-ID-F | GGCATATGTGTGCCACGATCG | Identification *FgASC1*  deletion transformants |
| FGSG_09870-ID-R | CTCTTCTTCTCGAGATCGAAG |  |
| FGSG_02105-P1 | TCTGACGGTCGTGACTGGTTG | Upstream fragment of *FgApm4* |
| FGSG_02105-P2 | CAAAATAGGCATTGATGTGTTGACCTCCGCCACTCATCTCACTTCCTC |  |
| FGSG_02105-P3 | CTCGTCCGAGGGCAAAGGAATAGAGTAGGAGGTAGAACATGTACGGCG | Downstream fragment of *FgApm4* |
| FGSG_02105-P4 | TATCGCGCTGACGTGGGATGG |  |
| FGSG_02105-ID-F | AAGTCATCCTGTTGCTCGTCG | Identification *FgApm4*  deletion transformants |
| FGSG_02105-ID-R | CACGGATAGAGGCAAGTTTCA |  |
| FGSG_01316-P1 | CAAAATAGGCATTGATGTGTTGACCTCCATGGGGCTGCCAGGGTTTCGAT | Upstream fragment of *FgAbp1* |
| FGSG_01316-P2 | CTTGAGGGAGCTTTGAGAGATG |  |
| FGSG_01316-P3 | CTCGTCCGAGGGCAAAGGAATAGAGTAGCATTGATTGGGTTCAACTTACAA | Downstream fragment of *FgAbp1* |
| FGSG_01316-P4 | CAATCATTCAAACGCAGTCGCAA |  |
| FGSG_01316-ID-F | TCGACGTGACTTCTGCTTCTC | Identification *FgAbp1*  deletion transformants |
| FGSG_01316-ID-R | GTTGGGCTGTCGGTTGTATG |  |
| Myo1-S2-LF | ATCTCGAGCATACTACAACATGAAGGCAT | Upstream fragment of plasmid pSilent-FgMYO1 |
| Myo1-S2-LR | ATAAGCTTGATCTTGCTCAGGCTGAG |  |
| Myo1-S2-RF | ATAGATCTGATCTTGCTCAGGCTGAG  ATGGTACCCATACTACAACATGAAGGCAT | Downstream fragment of plasmid pSilent-FgMYO1 |
| Myo1-S2-RR |  |  |
| Pzear-FgMYO1-P1 | CGAGGCGATGTATTATGGAC | Upstream fragment of *Fg* MYO1 promoter |
| Pzear-FgMYO1-P2 | CAAAATAGGCATTGATGTGTTGACCTCCACTCTCCCATCTCCCCTGTC |  |
| Pzear-FgMYO1-P3 | GATCCAGACCAGAGAGAACGAAAGTAACCATGGTACTGTATAAACTGTCCCGT | Downstream fragment of *Fg* MYO1 promoter |
| Pzear-FgMYO1-P4 | AAAAGGTTGCTGTAGATGGC |  |
| Pzear-FgMYO1-ID-F | GTGCCTATTTGTTTGTTGTC | Identification *Fg* MYO1 promoter deletion transformants |
| Pzear-FgMYO1-ID-R | CGCTTTGCCGCCTCTGTCTT |  |
| Myo1E420K-mutatin-F | ACTCGTCACGATTCGGAAAGT | Identification *Fg*Myo1-mutatin |
| Myo1E420K-mutatin-R | TAGGTGTCCATTGGATCTGCT |  |
| Tri1-GFP-F | ACTCACTATAGGGCGAATTGGGTACTCAAATTGGTTTTGTGAGTAGGCCTCATA | A pair of PCR primers to amplify *Tri1* fragments used for construction of the Tri1-GFP, vector under its own promoter |
| Tri1-GFP-R | CACCACCCCGGTGAACAGCTCCTCGCCCTTGCTCACGTCATCCTGTACCAATTCCAATCG |  |
| Tri4-RFP-F | ACTCACTATAGGGCGAATTGGGTACTCAAATTGGTTGGATACGATTGATAAAGTTTCT | A pair of PCR primers to amplify *Tri4* fragments used for construction of the Tri4-RFP, vector under its own promoter |
| Tri4-RFP-R | CATGAACTCCTTGATGACGTCCTCGGAGGAGGCCATCAAAGCCTTGAGAACCTTGACT |  |
| H1-RFP-F | ACTCACTATAGGGCGAATTGGGTACTCAAATTGGTTCTCGGAATAACACCGAAACTAC | A pair of PCR primers to amplify H1fragments used for construction of the H1-RFP, vector under its own promoter |
| H1-RFP-R | CATGAACTCCTTGATGACGTCCTCGGAGGAGGCCATCGCCTTGGCAGCAGCAGCAGCA |  |
| MYO1-GFP-F | ACTCACTATAGGGCGAATTGGGTACTCAAATTGGTTTGAGCAAGGTTTAGTAGTC | A pair of PCR primers to amplify *FgMYO1* fragments used for construction of the FgMYO1-GFP, vector under its own promoter |
| MYO1-GFP-R | CACCACCCCGGTGAACAGCTCCTCGCCCTTGCTCACCCAGTCATCGTCGTCTTCC |  |
| MYO1-RFP-F | ACTCACTATAGGGCGAATTGGGTACTCAAATTGGTTACATCAAACGTTGAAGAACG | A pair of PCR primers to amplify *FgMYO1* fragments used for construction of the FgMYO1-RFP, vector under its own promoter |
| MYO1-RFP-R | CATGAACTCCTTGATGACGTCCTCGGAGGAGGCCATCCAGTCATCGTCGTCTTCCTTC |  |
| MYO1-Flag-F | CTATAGGGCGAATTGGGTACTCAAATTGGTT ACATCAAACGTTGAAGAACG | A pair of PCR primers to amplify *FgMYO1* fragments used for construction of the FgMYO1-Flag, vector under its own promoter |
| MYO1- Flag-R | CTTTATAATCACCGTCATGGTCTTTGTAGTCCCAGTCATCGTCGTCTTCCTTC |  |
| Tri1-Flag-F | CTATAGGGCGAATTGGGTACTCAAATTGGTTTTGTGAGTAGGCCTCATA | A pair of PCR primers to amplify *FgTri1* fragments used for construction of the Fg *Tri1*-Flag, vector under its own promoter |
| Tri1-Flag-R | CTTTATAATCACCGTCATGGTCTTTGTAGTCGTCATCCTGTACCAATTCCAATCG |  |
| Actin-RFP-F | ACTCACTATAGGGCGAATTGGGTACTCAAATTGGTTGCCAGTCGGAATGACTGGGTGA | A pair of PCR primers to amplify *Fg* Actin fragments used for construction of the FgActin-RFP, vector under its own promoter |
| Actin-RFP-R | CATGAACTCCTTGATGACGTCCTCGGAGGAGGCCATGAAGCACTTGCGGTGAACGA |  |
| ASC1-RFP-F | ACTCACTATAGGGCGAATTGGGTACTCAAATTGGTTGATGCTCTCAGACGACTGGT | A pair of PCR primers to amplify *Fg* Prk1 fragments used for construction of the Fg ASC1-RFP, vector under own promoter |
| ASC1-RFP -R | CATGAACTCCTTGATGACGTCCTCGGAGGAGGCCATTGCCCTCGACATGACACCCCA |  |
| AurJ-RFP-F | ACTTGACTTACTTCTTGAACGATGGGTTCAATTTCTTCTCCAT | A pair of PCR primers to amplify *Fg* AurJ fragments used for construction of the Fg AurJ RFP, vector under  own promoter |
| AurJ-RFP-R | CATGAACTCCTTGATGACGTCCTCGGAGGAGGCCATGTCATCCTGTAAGCTGATGGTCA |  |
| Pex3-GFP-F | ACTCACTATAGGGCGAATTGGGTACTCAAATTGGTT ACTCACTATAGGGCGAATTGGGT | A pair of PCR primers to amplify Fg Pex3 fragments used for construction of the FgPex3-GFP, vector under its own promoter |
| Pex3-GFP-R | CACCACCCCGGTGAACAGCTCCTCGCCCTTGCTCAC CACCACCCCGGTGAACAGCTCC |  |
